# Identification of a Ste20-like kinase-dependent signaling network orchestrating cytoskeletal and synaptic development of cortical neurons

**DOI:** 10.1186/s12964-026-03250-y

**Published:** 2026-10-02

**Authors:** Anne Quatraccioni, Mark E. Graham, Marc Sylvester, Robert Hardt, Ashley J. van Waardenberg, Albert J. Becker, Susanne Schoch

**Affiliations:** 1 Institute of Cellular Neurosciences II, Medical Faculty, University of Bonn, Bonn, Germany; 2 Synapse Proteomics, Children’s Medical Research Institute, The University of Sydney, Westmead, Australia; 3 Core Facility Analytical Proteomics, Institute for Biochemistry and Molecular Biology, University of Bonn, Bonn, Germany; 4 Synapse PTY LTD, Cairns, Australia

**Keywords:** Kinase signaling, Protein–protein interactions, Affinity purification-mass spectrometry, Phosphoproteomics, Germinal center kinases

## Abstract

**Background:**

The Ste20-like kinase (SLK) is a serine/threonine kinase that regulates cytoskeletal organization, apoptosis, and cell polarity in non-neuronal cells. In neurons, SLK guides dendrite morphogenesis and the stability of inhibitory synapses, thereby controlling excitation-inhibition balance through parvalbumin interneuron function. Almost complete loss of SLK in dysmorphic neurons of epileptogenic cortical dysplasias implicates a function of this kinase in neurodevelopmental disorders. However, its neuronal signaling mechanisms remain poorly understood.

**Methods:**

To address this knowledge gap, we combined an affinity purification-mass spectrometry screen for candidate SLK-interacting proteins with quantitative phosphoproteomics after SLK knockdown to identify potential SLK interactors and the SLK-dependent phosphorylation landscape in cortical neurons.

**Results:**

Across the two datasets, we identified hundreds of candidate SLK-binding proteins and more than 1,000 regulated phosphosites after SLK knockdown. The datasets converged on cytoskeletal, synaptic, and signaling proteins involved in actin and microtubule regulation, cell adhesion, and synaptic stability. We identified two germinal center (GCK)-family kinases, MINK1 and TNIK, as direct SLK substrates at the activation-loop threonine (T181), suggesting that SLK may act upstream of a GCK kinase pathway implicated in dendritic development. Functionally, SLK knockdown also altered spontaneous network activity, changed the phosphorylation pattern of KCNQ2, and increased the sensitivity of neuronal networks to KCNQ2 blockade.

**Conclusions:**

These data position SLK within a broad neuronal signaling network that links cytoskeletal and synaptic regulation to kinase signaling and neuronal excitability. While the effects on KCNQ2 appear to be indirect, MINK1 and TNIK emerge as direct SLK substrates.

**Supplementary Information:**

The online version contains supplementary material available at https://doi.org/10.1186/s12964-026-03250-y.

## Introduction

Phosphorylation tunes protein activity, localization, stability, and interactions, and thereby shapes cellular behavior [1, 2]. In the nervous system, kinases play crucial roles in regulating axon and dendrite development, including dendrite outgrowth, branching, stabilization, and synapse formation, which determine neuronal connectivity and function [3]. Among these, STE20-family kinases, which comprise germinal center kinases (GCKs) and p21-activated kinases (PAKs), regulate neuronal morphology and signaling [4–6]. PAKs mediate signaling downstream of the Rho-family GTPases and are required for neuronal polarization, axon development, dendritic arborization, and dendritic spine morphogenesis. PAK signaling is involved in synapse formation, synaptic plasticity, and cognitive function, while dysregulated PAK activity has been linked to neurodevelopmental disorders including intellectual disability (ID) and autism spectrum disorders (ASD) [4]. Members of the GCK subfamily regulate neuronal architecture through pathways that control cytoskeletal organization and dendritic and synaptic development [7–9].

The Ste20-like kinase SLK belongs to the GCK-V subfamily of STE20 kinases and was initially characterized in non-neuronal cells, where it controls focal adhesion turnover, actin remodeling, cell migration, and apoptosis through interactions with microtubule- and actin-associated proteins [10–14]. Increasing evidence indicates that SLK also plays an essential role in the developing nervous system by regulating dendritic branching and inhibitory synapse stability [15]. Loss of SLK reduced dendritic branching of higher-order dendrites and selectively decreased the density of inhibitory synapses, without affecting the density of excitatory synapses [15]. These morphological alterations result in changes in neuronal function, as the frequency of miniature inhibitory postsynaptic currents (mIPSCs) and the amplitude of evoked inhibitory postsynaptic currents (IPSCs) are reduced in SLK knockdown (KD) neurons, shifting the excitation-inhibition balance of these cells [15, 16]. Further, SLK expression is reduced in dysmorphic neurons of human focal cortical dysplasias, a form of pediatric epilepsy [15]. This finding, combined with the observation that SLK regulates the excitation-inhibition balance, suggests that SLK dysfunction may contribute to epileptogenesis [16]. Notably, SLK variants have now been reported in patients with neurodevelopmental disorders, underscoring the clinical relevance of SLK signaling [17–19].

Despite these advances, the molecular steps linking SLK to neuronal structure and excitability remain poorly defined, as neuronal substrates and signaling partners of SLK are largely unknown. Here, we combined affinity purification-mass spectrometry (AP-MS) using brain lysate with quantitative phosphoproteomics in cultured cortical neurons. The two approaches identified candidate SLK-associated proteins and phosphorylation events across synaptic, cytoskeletal, adhesion, and signaling proteins. We identified two GCK-family kinases, MINK1 and TNIK, as direct SLK substrates. Their phosphorylation at threonine 181 in the activation loop suggests that SLK may act upstream of a pathway regulating dendritic development. In summary, this study provides the first molecular map of SLK-dependent signaling in neurons, pointing to SLK as a central regulator of dendritic architecture and synaptic circuit formation.

## Methods

### Ethical approvals

All animal procedures were planned and performed to minimize pain and suffering, and to reduce the number of animals used in accordance with European (guidelines of the European Parliament and of the Council on the protection of animals used for scientific purposes, European Directive (2010/63/EU)) and federal law (TierSchG, TierSchVersV) and approved by the state of North Rhine-Westphalia (Landesamt für Verbraucherschutz und Ernährung, LAVE, Germany). Animal studies are reported in compliance with the ARRIVE guidelines.

### Preparation of primary cultured neurons

For the preparation of primary cortical neurons, embryonic day (E) 16–19 mouse embryos were decapitated. The cortex was microdissected into cold HBSS, and cells were dissociated by adding 0.25% Trypsin. After a 20-min incubation at 37 °C, cells were washed and plated. After 24 h, the medium was replaced by Basal Medium Eagle (BME) supplemented with 2% B-27, 1% fetal bovine serum (FBS), 0.45% glucose, and 0.5 mM L-glutamine.

### Multi-electrode array recordings

For multi-well multi-electrode array (MEA) recordings, primary cortical neurons were seeded at a density of 40,000 cells per well in serum-free Neurobasal™ medium supplemented with 2% B-27™ (Gibco) and 0.8 mM L-glutamine in poly-D-lysine-coated CytoView MEA 24-well (Axion Biosystems) plates. Cells were transduced with adeno-associated viruses (rAAV2-1-U6-CMV-hrGFP, rAAV2-1-U6-shSLK-CMV-hrGFP) at an MOI of 500 on day in vitro (DIV) 4 and kept at 37 °C in 5% CO2. The activity of the neurons was measured in a Maestro Edge (Axion Biosystems) at DIV15, 18, and 21 for 10 min in neural spike mode, using AxiS Version 3.11.1.22 with a sampling frequency of 12.5 kHz and digital low-pass (3 kHz) and high-pass (200 Hz) filters. Spikes were detected by the adaptive threshold crossing method using 6 × the standard deviation. The weighted mean firing rate was calculated as the mean firing rate of active electrodes, i.e., electrodes with > 5 spikes/min. A burst was defined as a minimum of 5 spikes with a maximum inter-spike interval of 100 ms. Only wells with a minimum of 14 active electrodes on DIV21 were included in the analysis. Cell viability was determined using the alamarBlue assay (Invitrogen) according to the manufacturer’s instructions.

### Generation of constructs

To knock down SLK expression, a short hairpin RNA (shRNA) against SLK was expressed in a lentiviral expression vector (pLVTHM-Ef1α-mRFP-H1-shSLK; Control: pLVTHM-Ef1α-mRFP-H1-MCS) or in an adeno-associated viral expression vector (pAAV-U6-shSLK-CMV-hrGFP, Control: pAAV-U6-MCS-CMV-hrGFP). To overexpress SLK, SLK was cloned into a lentiviral expression vector (pLenti-Ef1α-mCherry-T2A-SLK-HA; Control: pLenti-Ef1α-mCherry-T2A-HA). CRMP1, SHISA7 (194–558), MINK1, and TNIK were amplified from mouse cDNA and cloned into the pEGFP-C1 (Clontech) vector using SacI and SalI restriction sites. For in vitro kinase reactions, CRMP1, MAP1B (1807–1982), MAP1B (402–678), MAP1B (171–793), MAP2 (1582–1723), MINK1 (140–316), NAV1 (492–672), NAV1 (39–283), NEFH (412–807), SHISA7 (211–345), and TNIK (140–316) were amplified from mouse cDNA and cloned into the pGEX-GST vector using XbaI and SalI restriction sites.

### In vitro kinase reactions

Plasmids encoding glutathione-S-transferase (GST)-tagged proteins were transformed into BL21 competent E. coli and grown in 500 ml LB medium supplemented with antibiotics at 37 °C under constant shaking. After reaching an optical density OD = 0.5–0.7, protein expression was induced by adding 0.1 mM Isopropyl β-D-1-thiogalactopyranoside (IPTG) for four hours. Pelleted bacteria were resuspended in lysis buffer (PBS + 1 mg/ml lysozyme (Roth) + protease inhibitor (Roche)) and sonicated for 2 min in intervals of 10 s at 40% amplitude using a Sonopuls HD2200.2 sonicator (MS73, Bandelin). Debris was removed by a one-hour centrifugation step at 4 °C, and the protein lysate was incubated with 1 ml of 50% glutathione-agarose beads (Sigma-Aldrich) for 2 h. To remove nonspecific binding, beads were washed four times and then resuspended in buffer to yield a 50% slurry.

10 µg of the GST-tagged protein, 50 µM UltraPure ATP, and 100 ng of SLK were mixed in 1 × Kinase Assay Buffer III (SLK kinase enzyme system, Promega), incubated for 30 min at 30 °C, and boiled after adding Laemmli Buffer (375 mM Tris-HCl, 12% SDS, 30% Glycerol, pH 6.8, 10% β-mercaptoethanol) at 95 °C for 10 min.

### Adeno-associated virus and lentivirus preparation

Human embryonic kidney (HEK) 293T cells were grown in Dulbecco’s Modified Eagle Medium (DMEM)-GlutaMAX supplemented with 10% FBS and 300 µg/ml G418. For lentiviral preparation, 3 × 10^6^ cells were seeded onto 100 mm cell culture dishes in DMEM-GlutaMAX with 10% FBS. For transfection with a 2nd-generation lentiviral packaging system, 7.5 µg packaging plasmid (psPAX2, gift from Didier Trono, Addgene #12,260), 5 µg of an envelope-expressing plasmid (pMD2.G, gift from Didier Trono, Addgene #12,259), and 4 µg of the expression plasmid were combined with 40 µl of GenJet Plus Reagent in 3 ml DMEM-GlutaMAX without FBS supplement and incubated for 20 min before adding it to the cells. 12 h later, cells were washed, and the medium was exchanged to DMEM-GlutaMAX supplemented with 10% FBS. 96 h after transfection, the supernatant of the transfected cells was filtered through 0.45 µm PVDF filters, and phase separation was performed by adding 300 µl of 60% OptiPrep Density Gradient Medium, followed by 2 h of ultracentrifugation at 24,000 rpm at 4 °C with an SW-Ti32 swinging bucket. After a second ultracentrifugation with TBS-5 (50 mM Tris–HCl, 130 mM NaCl, 10 mM KCl, 5 mM MgCl_2_), the pelleted virus was resuspended in 50 µl of TBS-5, and 5 µl was used for transduction of primary cortical neurons.

Adeno-associated viruses were prepared as described before [20]. The genomic titer of the viruses was determined by quantitative PCR using the SYBR Green assay.

### Sample preparation for phosphoproteomics

For whole-cell phosphoproteomics analysis, cultured cortical neurons were plated at a density of 3 × 10^5^ cells per well into poly-D-lysine-coated 6-well cell culture plates in BME supplemented with 2% B-27, 1% FBS, 0.45% glucose, and 0.5 mM L-glutamine. At DIV4–6, neurons were transduced with lentivirus and further grown at 5% CO_2_ at 37 °C until harvesting at DIV14-15. Neurons were washed in PBS and harvested in lysis buffer (2% SDS, 50 mM Tris–HCl, pH 7.4, 2 mM ethylene glycol-bis(β-aminoethyl ether)-N,N,N′,N′-tetraacetic acid (EGTA), 2 mM ethylenediaminetetraacetic acid (EDTA), cOmplete EDTA-free Protease Inhibitor Cocktail, 2 mM phenylmethylsulfonyl fluoride (PMSF), 5 mM NaF, 2 mM beta-glycerophosphate, Phosphatase Inhibitor Cocktail II (Calbiochem), and PhosSTOP (Roche)). Lysates were frozen on dry ice, incubated at 85 °C for 10 min, and sonicated for 9 × 10 s. An aliquot was used for immunoblotting, and the remaining lysate was lyophilized.

### Affinity purification (AP)

For the AP-MS experiments, HEK293T cells were cultured in DMEM supplemented with 10% FBS and 1% Penicillin–Streptomycin at 37 °C in 5% CO_2_. For transfection, 1.5 × 10^6^ cells were seeded into 100 mm cell culture dishes. 20 h later, the medium was changed to Iscove’s Modified Dulbecco’s Medium (IMDM) supplemented with 5% FBS, and 4 h later, cells were transfected with 10 µg of plasmid using calcium phosphate transfection. The following day, the medium was replaced with DMEM supplemented with FBS and Penicillin–Streptomycin, and 48 h after transfection, cells were harvested in lysis buffer (50 mM HEPES, 150 mM NaCl, 1% Triton X-100, cOmplete EDTA-free protease inhibitor, pH 7.4). Cell lysis was performed at 4 °C for 1 h, and debris was pelleted by centrifugation. The supernatant of HEK293T cell lysates was added to 25 µl of pre-washed HA magnetic beads (Pierce, Thermo Fisher Scientific) and incubated for 2 h at 4 °C. Subsequently, the bead-bound proteins were washed twice with Wash Buffer 1 (50 mM HEPES, 100 mM NaCl, 0.5% Triton X-100, pH 7.4), twice with Wash Buffer 2 (50 mM HEPES, 500 mM NaCl, 0.5% Triton X-100, pH 7.4), twice with Wash Buffer 1, and twice with DPBS before they were incubated with the whole-brain lysate. The preparation of whole-brain lysate was performed as described before [21] by isolating brains from 15–30-week-old C57BL/6 mice. 5 ml of lysis buffer (100 mM Tris–HCl, 150 mM NaCl, 1 mM EDTA, 1% TritonX-100, 1% sodium deoxycholate, 1% N-octyl-β-D-glucopyranoside; pH 6.5, supplemented with protease inhibitor) was added per gram of brain weight, and the brain was mechanically dissected using a Polytron homogenizer and tissue grinder. After a 3 h incubation at 4 °C on a rotator, cell debris was removed by centrifugation for 15 min at 4 °C with 7,000 g and ultracentrifugation for 30 min at 4 °C with 70,000 g. The protein-containing supernatant was incubated with SLK bound to magnetic beads overnight at 4 °C. To remove nonspecific binding, the beads were washed 5 times with Wash Buffer 3 (25 mM Tris, 150 mM NaCl, 1 mM EDTA, 1% Triton X-100, 5% Glycerol, pH 7.4) and then boiled with Laemmli Buffer.

### SDS polyacrylamide gel electrophoresis (SDS-PAGE) for mass spectrometry

Protein samples from the AP and in vitro kinase experiments were separated on NuPAGE 4–12% Bis–Tris polyacrylamide gels in NuPAGE MOPS SDS-running buffer (Invitrogen) and stained with colloidal Coomassie Brilliant Blue (RotiBlue, Roth). Specific protein bands were excised from the gel and subjected to mass spectrometry. For the analysis of all proteins isolated by AP, gels were run only briefly, and a gel piece containing the non-separated proteins was isolated.

### Co-immunoprecipitation

For co-immunoprecipitation experiments in HEK293T cells, pLenti-Ef1α-mCherry-T2A-SLK-HA and the respective EGFP-tagged protein were transfected in molar ratios of the plasmid size. After lysing the cells as described above, proteins were incubated with 30 µl of GFP-Trap magnetic beads (ChromoTek) for 2 h, followed by 5 washing steps with Wash Buffer 3. Samples were boiled in Laemmli Buffer and analyzed by immunoblotting.

### Immunoblotting

For immunoblotting, proteins were separated by SDS-PAGE and transferred to a nitrocellulose membrane overnight. Nonspecific protein binding was blocked by incubating the membrane for 1 h with 2% fish gelatin in PBS. Primary antibodies (SLK rabbit, MBS8233912, MyBioSource, 1:1000; GFP mouse, ab38689, abcam, 1:1000; β-actin mouse, ab6276, abcam, 1:5000) were diluted in blocking buffer and incubated for 2 h at room temperature. After washing the membrane with PBST (PBS + 0.1% Tween) three times, membranes were incubated with secondary antibodies IRDye 800CW goat α-rabbit (926–32,211, LI-COR Odyssey, 1:10,000), IRDye 680RD goat α-mouse (926–68,070, LI-COR Odyssey, 1:10,000), or IRDye 680LT donkey α-mouse (926–68,022, LI-COR Odyssey, 1:10,000) for 45 min, followed by 3 washing steps with PBST. Membranes were imaged by an Odyssey Imaging System (Odyssey DLx, LI-COR) and analyzed with ImageStudio Software (LI-COR).

### Mass spectrometry (MS)

#### LC–MS/MS of phosphoproteins

Lyophilized neuronal samples were digested, labeled with TMT10plex reagent, combined, and enriched for phosphopeptides. After fractionation by hydrophilic ion chromatography, the samples were analyzed by Liquid Chromatography (LC)-MS/MS and statistically compared using the same methods and equipment as described previously [22].

#### Peptide preparation for AP-MS and in vitro kinase reactions

Gel slices were subjected to gel digestion [23, 24]. In brief, slices were washed consecutively with water, 50% acetonitrile (ACN), and 100% ACN. Proteins were reduced with 20 mM Dithiothreitol (DTT) in 50 mM ammonium bicarbonate and alkylated with 40 mM acrylamide (in 50 mM bicarbonate). The slices were washed again and dehydrated with ACN. Proteolysis was performed with 330 ng trypsin (sequencing grade, Promega, Mannheim, Germany) at 37 °C overnight. The peptide extracts were dried in a vacuum concentrator and stored at −20 °C.

#### LC–MS analysis for AP-MS

Dried peptides were dissolved in 10 µl 0.1% formic acid (solvent A). Peptide separation was performed on a Dionex Ultimate 3000 RSLC nano HPLC system (Dionex GmbH, Idstein, Germany). The autosampler was operated in μl-pickup mode. 2 µl were injected onto a C18 analytical column (350 mm length, 75 µm inner diameter, ReproSil-Pur 120 C18-AQ, 1.9 µm). Peptides were separated during a linear gradient from 5% to 35% solvent B (90% ACN, 0.1% FA) at 300 nl/min within 60 min. The nano HPLC was coupled online to an Orbitrap Fusion Lumos mass spectrometer (Thermo Fisher Scientific, Bremen, Germany). The data-dependent acquisition was performed on ions between 330 and 1600 m/z scanned in the Orbitrap detector every 2 s with a resolution of 120,000 (gain control target 400,000). Polysiloxane (*m/z* 445.12002) was used for internal calibration. In a top-speed method, peptide ions were subjected to higher energy collision-induced dissociation (HCD: 1.0 Da isolation, threshold intensity 25,000, collision energy 30%), and fragments were analyzed in the Orbitrap (*R* = 15,000) with gain control target 50,000 and maximum inject time 22 ms. Fragmented peptide ions were excluded from repeat analysis for 15 s. Raw data processing and analysis of database searches were performed with Proteome Discoverer software 2.5.0.400 (Thermo Fisher Scientific). Peptide identification was done with an in-house Mascot server version 2.6.1 (Matrix Science Ltd, London, UK) from Proteome Discoverer. MS2 data were searched against a database of common contaminants (cRAP of the Global Proteome Machine) and the murine SwissProt sequences of UniProt (2021_03, 17,089 sequences). Precursor ion *m/z* tolerance was 10 ppm; fragment ion tolerance was 20 mmu. Tryptic peptides with up to two missed cleavages were searched; propionamide was set as a static modification of cysteines, oxidation of methionine, and acetylation of protein N-termini as dynamic modifications. Mascot results were sent to the Percolator algorithm [25] version 3.05 as implemented in Proteome Discoverer 2.5. Spectra without high-confidence matches (q-value > 0.01) were sent to a second-round search with semi-specific enzyme cleavage, dynamic modification of cysteines with propionamide, and serines/threonines with phosphate. Abundances were calculated as the sum of unique peptides extracted ion chromatogram areas. No normalization was applied.

#### LC–MS analysis of in vitro kinase reaction

LC–MS analysis for in vitro kinase reactions was performed as described for LC–MS analysis for AP-MS samples, except for the following changes: To reduce phosphopeptide losses due to metal ion binding, the LC–MS system was passivated by three citrate injections [26] before samples were analyzed. C18 analytical columns of 350 mm length and 100 µm inner diameter were used. The data-dependent acquisition was performed on ions between 330 and 1600 m/z, scanned in the Orbitrap detector every 3 s with a resolution of 120,000, with gain control target 400,000 and maximum inject time of 50 ms. After higher-energy collision-induced dissociation (HCD: 1.0 Da isolation, threshold intensity 30,000, collision energy 27%), fragments were analyzed in the Orbitrap (*R* = 30,000) with a gain control target of 50,000 and a maximum inject time of 54 ms. In the case of the detection of a phospho-specific neutral loss (97.9769 Da), an additional electron-transfer/higher-energy collision dissociation (EThcD, 1.0 Da isolation, threshold intensity 30,000) fragment spectrum was acquired in the Orbitrap (*R* = 30,000, gain control target = 100,000, maximum inject time 54 ms). Fragmented peptide ions were excluded from repeat analysis for 20 s. Targeted MS analyses were performed essentially as above, with the following exceptions: peptides were separated in a 90 min gradient. MS1 spectra were recorded every 3 s with a resolution of 60,000. Selected precursors were fragmented as above, but with a maximum injection time of 100 ms. Data were analyzed with the help of Skyline software [27].

For shotgun data analysis, MS2 data were searched against a database of common contaminants (cRAP of the Global Proteome Machine) and a custom database containing the sequences of all used recombinant GST-tagged proteins. Precursor ion *m/z* tolerance was 10 ppm; fragment ion tolerance was 20 ppm. Tryptic peptides (Trypsin/P) with up to two missed cleavages were searched; propionamide was set as a static modification of cysteines, oxidation of methionine, phosphorylation of serine and threonine, and acetylation of protein N-termini as dynamic modifications. Spectrum confidence of Mascot results was assessed by the Target Decoy PSM Validator node (Selection: Concatenated, Target FDR strict/relaxed: 0.01/0.05). Furthermore, phosphorylation sites were localized using the IMP-ptmRS node applying default settings. Extracted ion chromatogram areas of peptides were calculated by the Minora Feature detector using default settings. Protein abundances were generated by summing up unique peptides, excluding phosphorylated peptides. Furthermore, abundances were normalized to the individual protein used in the respective experiment. Protein ratios were calculated by the pairwise-ratio-based method, and differentially regulated proteins/peptides were determined by Proteome Discoverer’s background-based t-test method. Finally, protein groups, proteins, peptide groups, and peptide isoform result tables were exported to the Microsoft Excel format and used for further data evaluation.

### Analysis and statistics

#### Bioinformatic analysis of phosphoproteomic data

MaxQuant output files were reprocessed using methods previously described in R version 3.5.1 [22]. Briefly, phosphosites were remapped to the same FASTA file used for MaxQuant processing, and peptide sequences were centered around each phosphorylated site (± 7 AA). All phosphopeptides with a site localization score greater than 0.75 (class I) for at least one site were then retained. For multi-phosphorylated peptides, a score of > 0.5 was required for additional sites in a peptide with a class I site. If a phosphosite or multi-phosphosite was measured multiple times across unique phosphopeptides, the median log_2_-transformed intensity was calculated for the phosphosite or phosphosite combination. Where phosphosites mapped to more than one unique protein identifier, a unique identifier was assigned in accordance with previously described annotation criteria [28]. Only complete data with no missing values in any of the replicates were used for analyses. Data were then normalized by quantile normalization [29], and unwanted variation was removed by surrogate variable analysis [30]. Samples were clustered via principal component analysis and hierarchical clustering, which were used to assess the impact of normalization and selection of the number of surrogate variables, k, for the removal of unwanted variation. For differential phosphorylation analysis, data were fit to a generalized linear model with Bayes shrinkage using limma version 3.36.5 [31]. p-values were corrected for multiple comparisons using the Benjamini and Hochberg method (adjusted p-value (padj)) [32].

#### KinSwing analysis

Analysis of kinase motifs and kinase-substrate matches was performed with KinSwingR version 1.1.4 (http://bioconductor.org/packages/KinSwingR/) as described before [22, 28]. In short, position weight matrices (PWMs) were modeled for 160 kinases with at least 10 published phosphopeptides, by dividing the log-likelihood ratio of the average frequency of amino acid residues at each position by the background frequency. For SLK, three additional PWMs (centered on S, T, or S/T combined phosphosites) were built based on previously published and curated substrates of SLK (Table S1) using the *buildPWM* function of KinSwingR. Each position in the kinase motif was modeled and visualized as a Shannon Entropy model using the *viewPWM* function of KinSwingR. Visualized motif amino acids were colored according to the Shapely scheme, which assigns brighter colors to polar amino acids and duller colors to non-polar amino acids.

Subsequent scoring of each kinase PWM to each measured phosphopeptide was performed using the *scoreSequences* function of KinSwingR. Kinase activity was inferred by determining the standardized weighted proportion of substrate matches to each kinase, and statistical significance was determined using network perturbations (*n* = 1000) of PWM: substrate edges.

#### Bioinformatic analysis of AP-MS data

For the AP-MS data, mass spectrometry output files from Proteome Discoverer were analyzed in R version 3.5.1. For the experimental condition, 25% missing data was allowed. Unannotated and contaminant proteins were removed before further analysis.

Where proteomic data were derived from excised bands, data were log_2_-transformed, missing values imputed by the left-censored missing data approach QRILC (quantile regression imputation of left-censored data; as implemented in imputeLCMD: https://cran.rstudio.com/web/packages/imputeLCMD/index.html), and log-ratios (between experimental and control conditions to preserve overall differences in protein quantity) normalized using cyclic loess normalization [33]. For the AP-MS experiment in which the whole lane was excised from the gel and analyzed by mass spectrometry, data were treated the same, with additional batch correction performed using ComBat. Statistical significance was determined using limma statistics and multiple hypothesis correction according to the Benjamini and Hochberg method as described above.

#### Annotation analysis

Proteins identified in the proteomic screens were analyzed for their localization and biological function using the Functional Annotation Tool of the DAVID Bioinformatic Resources 6.8 (threshold: 2, threshold EASE 0.1, padj: Benjamini correction) [34, 35] and SynGO search (GSEA settings: minimum gene count per term: 3; all annotations included) [36].

Enrichment analysis of the AP-MS data was visualized with STRING v12.0 [37] using the brain proteome as background [38]. The disease network was visualized in Cytoscape (v. 3.10.3) [39] after analyzing the AP-MS dataset with the Functional Annotation Tool of the DAVID Bioinformatic Resources 6.8 (Up-KW disease, threshold: 2, threshold EASE 0.1, padj: Benjamini correction) using the brain proteome as background [38].

The enrichment map for the phosphoproteomics data was visualized in Cytoscape (v. 3.10.3) [39] after analyzing the dataset with g:Profiler [40] using all detected phosphoproteins as a custom background (significance threshold: Benjamini–Hochberg FDR, gene set size 5–1000).

#### Statistics

The mean and standard error of the mean are depicted in the graphs if not stated otherwise. Significance was assessed using GraphPad Prism v.10.1.1 and noted as *: *p* < 0.05, **: *p* < 0.01, ***: *p* < 0.001, ****: *p* < 0.0001.

### Use of AI tools

During the preparation of this manuscript, Grammarly, DeepL, and ChatGPT by OpenAI were used for language editing (refinement of English grammar, typing errors, and improvement of sentence clarity). The authors take full responsibility for the final content and integrity of the manuscript.

## Results

### Identification of candidate SLK-interacting proteins and the neuronal SLK-dependent phosphoproteome

**Fig. 1:**
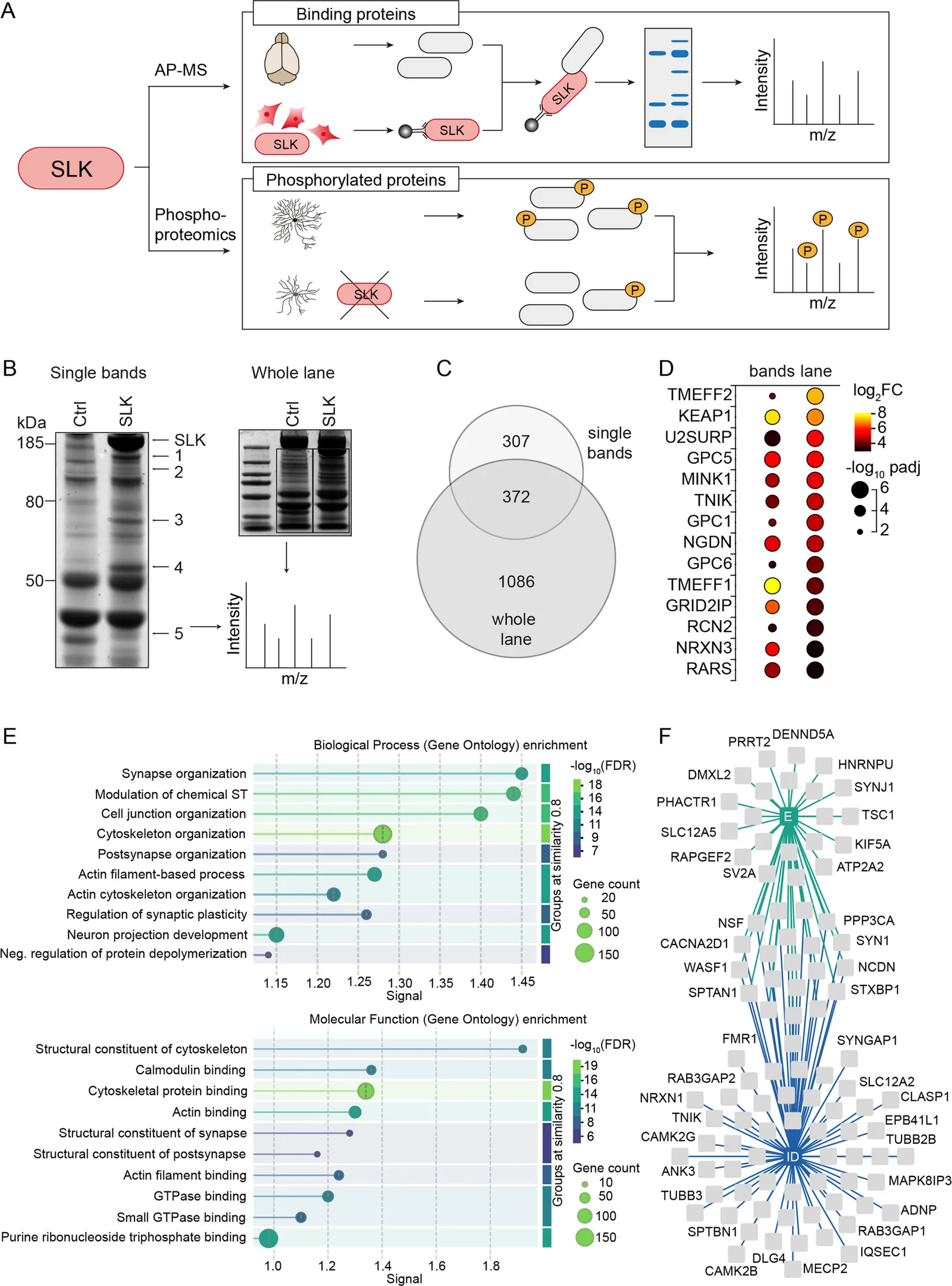
Identification of the SLK interactome and phosphoproteome by complementary proteomics screens. **A** Schematic of the experimental approach. Candidate SLK-binding proteins were identified by affinity purification (AP) from brain lysate followed by mass spectrometry. SLK-dependent phosphorylation events were identified by comparing the phosphoproteome of cultured control and SLK knockdown (KD) neurons after quantitative mass spectrometry. **B** Coomassie-stained SDS-PAGE of proteins co-immunoprecipitated with SLK or control (Ctrl). Bands present specifically in the SLK sample (numbered 1–5) and the whole lane, including all proteins, were excised and analyzed by mass spectrometry. *n* = 4 independent replicates for both approaches. **C** The Venn diagram illustrates that more than half of the proteins identified in the individual bands were also detected in the whole-lane analysis (padj < 0.05 & log_2_FC > 0). D Shared proteins that were identified in both band-specific and whole-lane analyses with a log_2_FC > 3 are depicted (padj < 0.05 & log_2_FC > 3). **E** Gene Ontology (GO) enrichment for proteins in the band-specific analysis (AP-MS single bands: padj < 0.05 and log_2_FC > 1). Functional annotation analysis with DAVID: Biological process and molecular function. ST: synaptic transmission. **F** Proteins identified in the band-specific AP-MS were enriched in proteins associated with epilepsy (E) and intellectual disability (ID) (AP-MS single bands: padj < 0.05 and log_2_FC > 1). Functional annotation analysis with DAVID: UP_KW_Disease

To investigate the signaling networks downstream of the kinase SLK, we applied two complementary proteomic approaches (Fig. 1A). First, affinity purification-mass spectrometry (AP-MS) was used to identify candidate proteins present in brain lysate that can interact with SLK. Second, phosphoproteomic analysis of control and SLK knockdown (KD) cortical neurons was applied to identify phosphorylation events that depend directly or indirectly on SLK.

For the interactome screen, SLK was expressed in HEK293T cells, purified by immunoprecipitation, and incubated with mouse whole-brain lysates. Proteins co-immunoprecipitated with SLK were separated by SDS-PAGE and visualized by Coomassie Blue staining. Bands present in the SLK but not in control samples were excised and analyzed by mass spectrometry (Fig. 1B). In parallel, entire lanes were analyzed to capture a broader set of candidate SLK-binding partners. A total of 372 (55%) out of 679 significantly enriched proteins detected in individual gel bands were identified in the global lane analysis (Fig. 1C), indicating substantial overlap between the two approaches. The incomplete overlap is most plausibly explained by the technical properties of LC–MS/MS. Single-band digests reduce sample complexity, thereby improving detection of low-abundance peptides, whereas the higher complexity of whole-lane digests can limit peptide identification because of ion suppression and stochastic precursor selection. Differences in digestion efficiency can add to this effect. The two approaches are therefore expected to overlap only partially, despite the underlying protein populations being largely identical.

Among the most highly enriched proteins in both screens were several proteins with known synaptic functions or localization, including Neurexin 3 (NRXN3), Glypicans (GPC1, 5, 6), the kinases Misshapen-like kinase 1 (MINK1) and Traf2 and NCK-interacting protein kinase (TNIK), and the transmembrane proteins Tomoregulin-1 and −2 (TMEFF1, TMEFF2) (Fig. 1D). Gene ontology (GO) analysis of proteins detected in the band-specific screen showed a significant enrichment for proteins involved in synapse organization, cell junction assembly, cytoskeletal dynamics, and GTPase-mediated signaling (Fig. 1E), which regulate diverse cellular functions such as gene expression, vesicle trafficking, and cytoskeletal organization [41]. Disease-related functional annotation analysis revealed that many candidates for SLK-interacting proteins were linked to epilepsy and intellectual disability (ID), two neurodevelopmental diseases associated with SLK dysfunction (Fig. 1F).

**Fig. 2:**
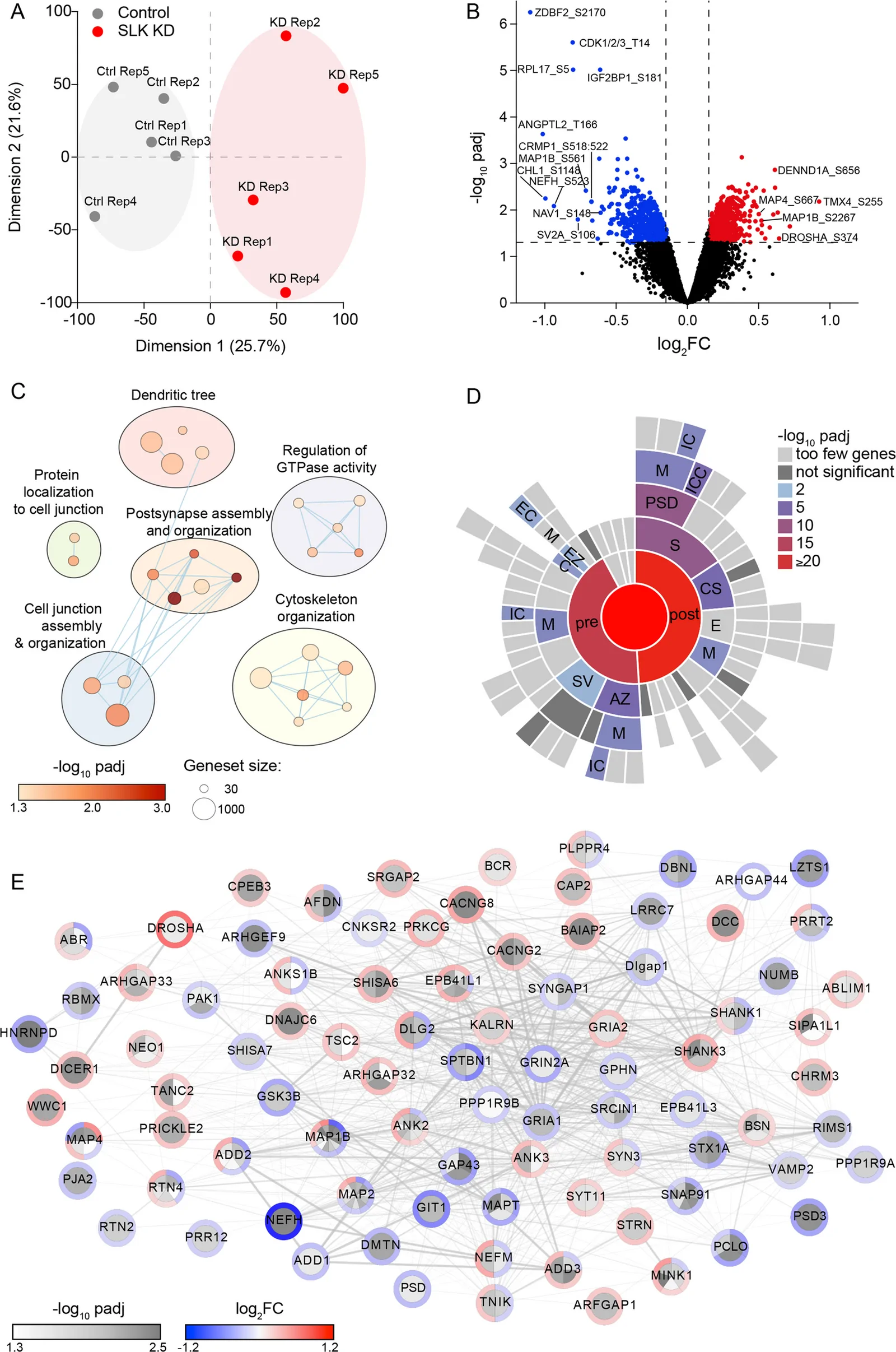
Phosphorylation profile of cultured cortical neurons after SLK knockdown. **A** Samples from control and SLK KD neurons are clearly separated in the principal component analysis (PCA) plot when analyzing the phosphorylation status of the neuronal proteins. *n* = 5 independent replicates. **B** The volcano plot depicts all detected phosphorylation sites. Sites exhibiting significantly reduced phosphorylation following SLK KD (padj < 0.05 and log_2_FC <−0.15) are shown in blue, whereas sites with significantly increased phosphorylation (padj < 0.05 and log_2_FC > 0.15) are shown in red. **C** Proteins with differentially phosphorylated sites are enriched in processes that are important for synapse and dendritic assembly, organization, and function. Depicted are all GO terms for cellular component and biological process with a gene set size of 5–1000 after analyzing all significantly differentially phosphorylated proteins (Phosphoproteomics: padj < 0.05). Each node represents a significantly enriched GO term, while edges connect GO terms that share a substantial proportion of associated genes. **D** SynGO analysis confirmed a selective enrichment for pre- and postsynaptic proteins with a specific emphasis on membrane proteins. AZ: active zone, C: cytosol, CS: cytoskeleton, E: endosome, M: membrane, IC: integral component, ICC: intracellular component, EC: extrinsic component, EZ: endocytic zone, PSD: postsynaptic density, S: postsynaptic specialization, SV: synaptic vesicle. **E** Network of postsynaptic density proteins that were differentially phosphorylated after SLK KD (Phosphoproteomics: padj < 0.05). Each ring represents a protein and is subdivided into segments corresponding to the individual phosphorylation sites. Individual segments indicate the log_2_-fold change (log_2_FC) for each phosphosite on that specific protein. The corresponding -log_10_ padj values are displayed within the ring.

As a protein kinase, SLK modulates other proteins through direct binding or by catalyzing phosphorylation, thereby extending the scope of its functional interactions. To identify the SLK-dependent phosphoproteome, cultured cortical neurons were transduced with lentiviruses expressing the fluorescent protein mRFP (control) alone or together with a short hairpin RNA against SLK (shSLK) to knock down SLK expression. Immunoblot analysis confirmed that the KD was efficient and decreased SLK expression by 93% (Fig. S1A). Lysates from control and SLK KD conditions were analyzed by LC–MS/MS, and the phosphorylation pattern of neuronal proteins was investigated. Principal component analysis (PCA) demonstrated a clear separation between the control and the SLK KD condition in the first dimension (Fig. 2A). Phosphorylation of 548 sites was significantly increased after SLK KD, indicating enhanced activity of other kinases or decreased activity of phosphatases, whereas the phosphorylation of 512 sites was significantly reduced following SLK KD. Proteins related to the cytoskeleton (e.g., MAP1B, NEFH, CRMP1) and to the synapse (SV2A, CHL1) were detected within the group showing reduced phosphorylation (Fig. 2B). Overall, significantly regulated phosphorylation sites were preferentially detected in proteins at the synapse or involved in cell adhesion, cytoskeletal and dendrite organization, and the regulation of GTPase activity (Fig. 2C, Fig. S1B). Subsequent SynGO analysis of the synaptic protein fraction identified an enrichment of pre- and postsynaptic proteins, with a specific emphasis on membrane-associated proteins located in the postsynaptic compartment (Fig. 2D, Fig. S1C). Proteins that were differentially phosphorylated after SLK KD and annotated as postsynaptic density proteins formed a tightly interconnected network (Fig. 2E) [42–45].

**Fig. 3:**
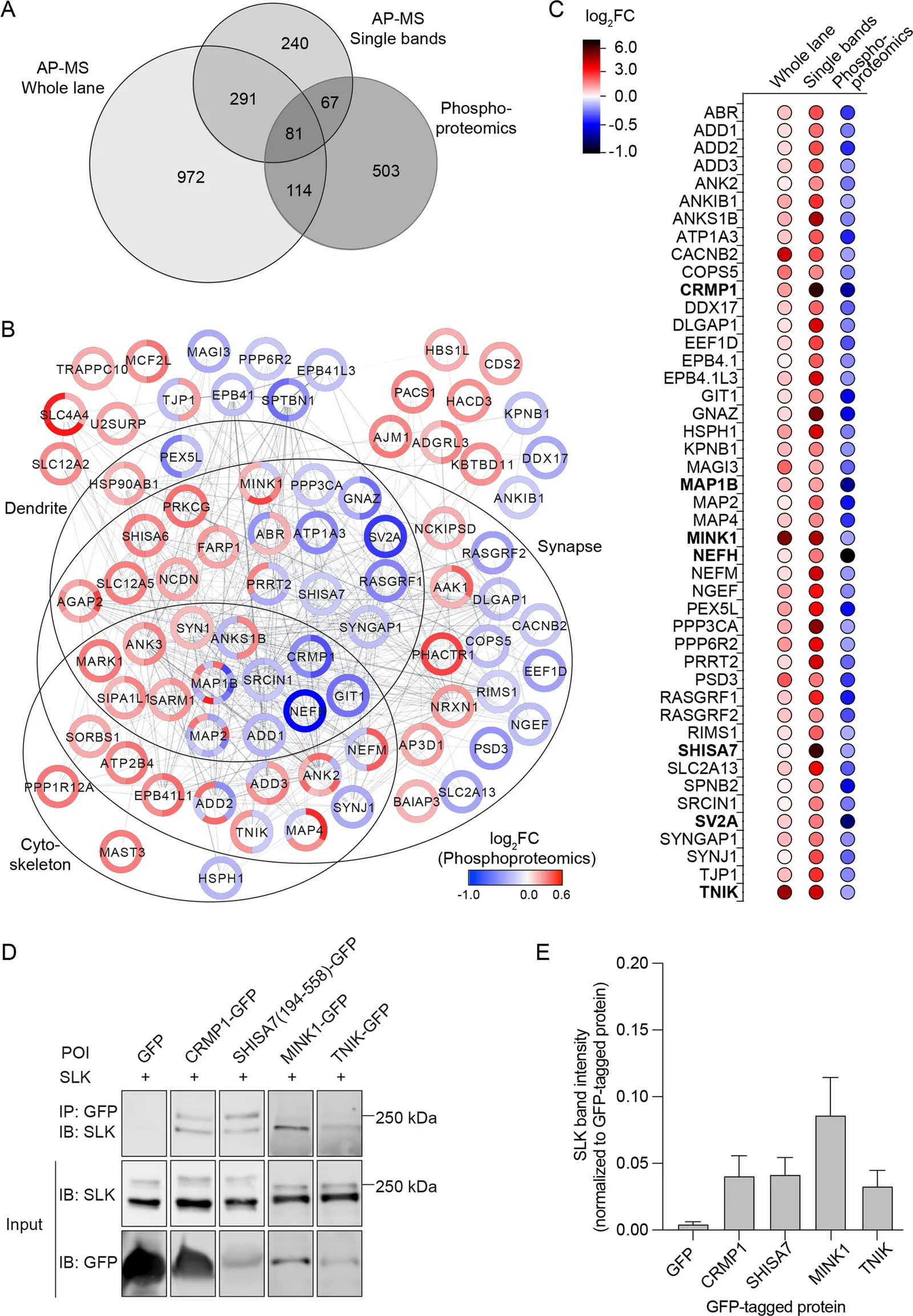
Integration of proteomic datasets. **A** The Venn diagram shows the number of proteins identified in each proteomic approach (Phosphoproteomics: padj < 0.05; AP-MS: padj < 0.05 & log_2_FC > 0). **B** Proteins identified in all three approaches are shown. The majority of these proteins are annotated as synaptic, dendritic, or cytoskeletal proteins (Phosphoproteomics: padj < 0.05 and AP-MS: padj < 0.05 & log_2_FC > 0). Individual segments of the rings denote distinct phosphorylation sites detected on the same protein, while the color of each segment encodes the log_2_-fold change (log_2_FC) for the respective phosphosite. **C** Proteins that exhibited reduced phosphorylation in SLK KD neurons and were also identified as binding partners of SLK in AP-MS experiments are shown, highlighting candidate direct substrates that interact with SLK (Phosphoproteomics: padj < 0.05 & log_2_FC < −0.15; AP-MS: padj < 0.05 & log_2_FC > 0). Candidates with the highest |log_2_FC| are marked in bold. **D** Candidate proteins identified in the proteomic screens were cloned as GFP-tagged proteins for inverse co-immunoprecipitation. GFP-tagged proteins were immunoprecipitated, and their potential to bind SLK was analyzed via immunoblotting. *n* = 4–5. IB: immunoblot, IP: immunoprecipitation, POI: protein of interest. **E** Quantification of co-immunoprecipitation results. The band intensity of SLK was normalized to the band intensity of the immunoprecipitated protein.

The two datasets reveal different aspects of SLK biology. The AP-MS dataset identifies candidate proteins that can associate with SLK under experimental conditions, whereas phosphoproteomics captures phosphorylation changes after SLK KD and their overlap may provide a focused set of candidates for validation. One-third of all proteins with differentially regulated phosphorylation sites were detected in at least one of the AP-MS approaches, whereas ~ 13–22% of co-immunoprecipitated proteins also showed significant phosphorylation changes (Fig. 3A). Proteins detected across all three datasets were enriched for synaptic, dendritic, and cytoskeletal functions (Fig. 3B). By focusing on potential SLK substrates, i.e., proteins identified as candidate SLK interactors and with reduced phosphorylation after SLK KD, we identified cytoskeletal proteins (MAP1B, MAP2), synaptic proteins (SV2A, GIT1, SHISA7), and the kinases MINK1 and TNIK (Fig. 3C). Among these proteins, the strongest reduction in phosphorylation in the phosphoproteomics dataset was detected for CRMP1, MAP1B, NEFH, and SV2A, while the proteins CRMP1, MINK1, TNIK, and SHISA7 were the strongest binding partners in the AP-MS datasets. To validate CRMP1, MINK1, TNIK, and SHISA7 as SLK-interacting proteins, we performed co-immunoprecipitation experiments by expressing the GFP-fused candidate proteins and HA-tagged SLK in HEK293T cells (Fig. 3D). All four proteins could be verified to bind to SLK with varying strength (Fig. 3D-E).

Together, our results indicate that SLK binds to and directly or indirectly influences the phosphorylation status of synaptic, cytoskeletal, and dendritic protein networks.

### SLK KD is associated with altered spontaneous network activity and increased sensitivity to KCNQ2 blockade

Synaptic proteins emerged as a prominent functional category in both the phosphoproteomics and the AP-MS dataset, highlighting synaptic signaling as a major process affected by SLK. SLK KD affects excitability of individual neurons in mice [15, 16], but it is unknown whether SLK KD alters excitability in neuronal networks in vitro and whether the altered synaptic phosphorylation patterns translate into a functional phenotype.

**Fig. 4:**
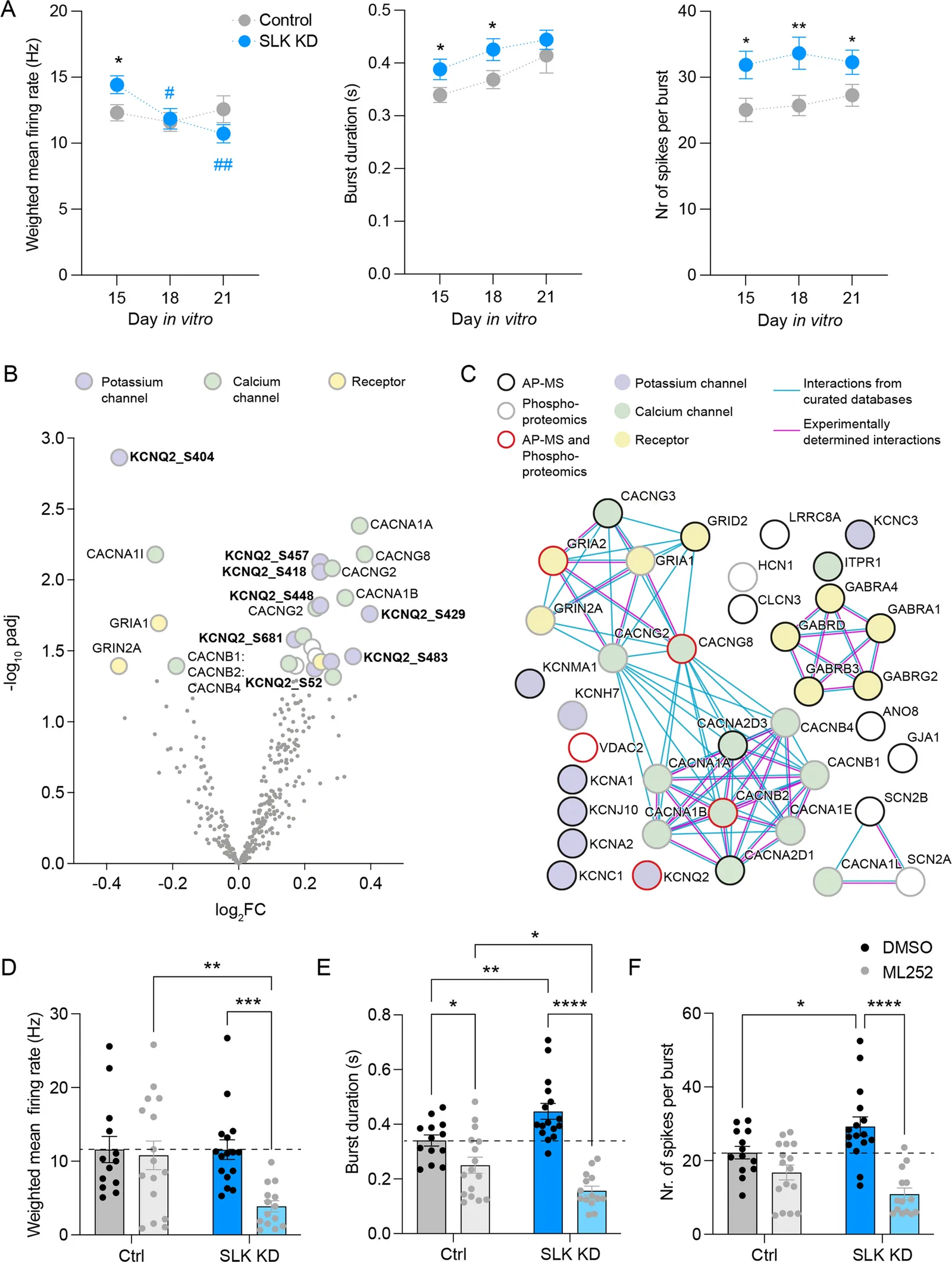
Altered network activity and sensitivity to KCNQ2 inhibition after SLK KD. **A** Spontaneous spiking activity was measured in control versus SLK KD neurons at DIV15, 18, and 21. SLK KD neurons had a higher weighted mean firing rate at DIV15, which declined by DIV21. Burst duration was slightly longer in SLK KD neurons at DIV15 and DIV18, and the number of spikes per burst was increased at all time points. *N* = 45–46 wells from 4 independent replicates. * significant difference compared to control. *#* significant difference compared to DIV15 of the same condition (two-way ANOVA with Holm-Šídák's multiple comparisons test). **B** The volcano plot depicts all phosphorylation sites that were detected in ion channel proteins. **C** The network of ion channel subunits was identified in the phosphoproteomics dataset and/or in at least one of the AP-MS datasets (Phosphoproteomics: padj < 0.05 & |log_2_FC| >0.15; AP-MS: padj < 0.05 & log_2_FC > 0). Nodes represent channel subunits, and connecting lines indicate known interactions. **D** Weighted mean firing rate in SLK KD neurons dropped upon application of the KCNQ2 inhibitor ML252, whereas control neurons were largely unaffected. *N* = 13–16 wells from 4 independent replicates. **E** Burst duration decreased in both groups after ML252 application, but the reduction was greater in SLK KD neurons. **F** ML252 reduced the number of spikes per burst in SLK KD neurons. D-F:*: *p* < 0.05, **: *p* < 0.01, ***: *p* < 0.001, ****: *p* < 0.0001 (two-way ANOVA with uncorrected Fisher's LSD)

To answer this question, neurons were transduced with an adeno-associated virus encoding a short hairpin RNA against SLK (shSLK), and spontaneous neuronal activity was measured by multi-electrode array (MEA) recordings at three time points (days in vitro (DIV) 15, 18, and 21) during synapse development and maturation [46]. At the earliest measured time point (DIV15), the weighted mean firing rate was significantly increased in SLK KD neurons and decreased until DIV21 (Fig. 4A, S2A-D). At the early stages of synapse maturation (DIV15 and 18), bursts in SLK KD neurons lasted longer in SLK KD compared to control neurons, and more spikes occurred within a burst for all time points (Fig. 4A, S2A-D). To exclude that the results were due to different seeding densities or neuronal survival, the number of active electrodes and the cell viability were determined and found to be unchanged between control and SLK KD neurons (Fig. S2E–F).

The prolonged burst duration and increased number of spikes per burst may reflect altered neuronal excitability resulting from widespread changes in the phosphorylation of proteins involved in ion channel regulation, synaptic transmission, and cytoskeletal organization. Such phosphorylation-dependent modulation could influence dendrite outgrowth, neurotransmitter release, synaptic connectivity, and also receptor function and ion channel gating. To determine whether SLK KD affected the phosphorylation status of ion channels and receptors, the phosphoproteomics dataset was filtered for this protein category. Several potassium and calcium channel subunits showed significant phosphorylation changes after SLK KD, suggesting that SLK KD may alter the molecular machinery underlying neuronal electrical signaling (Fig. 4B). Calcium and potassium channels were also detected as candidate SLK-interacting proteins in the AP-MS data (Fig. 4C). Among the ion-channel proteins detected in this analysis, the potassium channel subunit potassium voltage-gated channel subfamily KQT member 2** (**KCNQ2) exhibited the highest number of differentially phosphorylated sites (Fig. 4B).

To test whether SLK KD alters the dependence of neuronal network activity on KCNQ2 function, we examined the response of primary cortical neurons on DIV21 to the KCNQ2 blocker ML252 using MEA recordings. While the application of ML252 did not change the weighted mean firing rate of control neurons, it reduced the weighted mean firing rate of SLK KD neurons by more than half (Fig. 4D). In addition, the application of ML252 led to a greater reduction in the burst duration and the number of spikes per burst in SLK KD neurons (Fig. 4E-F). Thus, SLK-deficient neuronal networks show an increased sensitivity to pharmacological KCNQ2 inhibition.

### Substrate consensus motifs for SLK

**Fig. 5:**
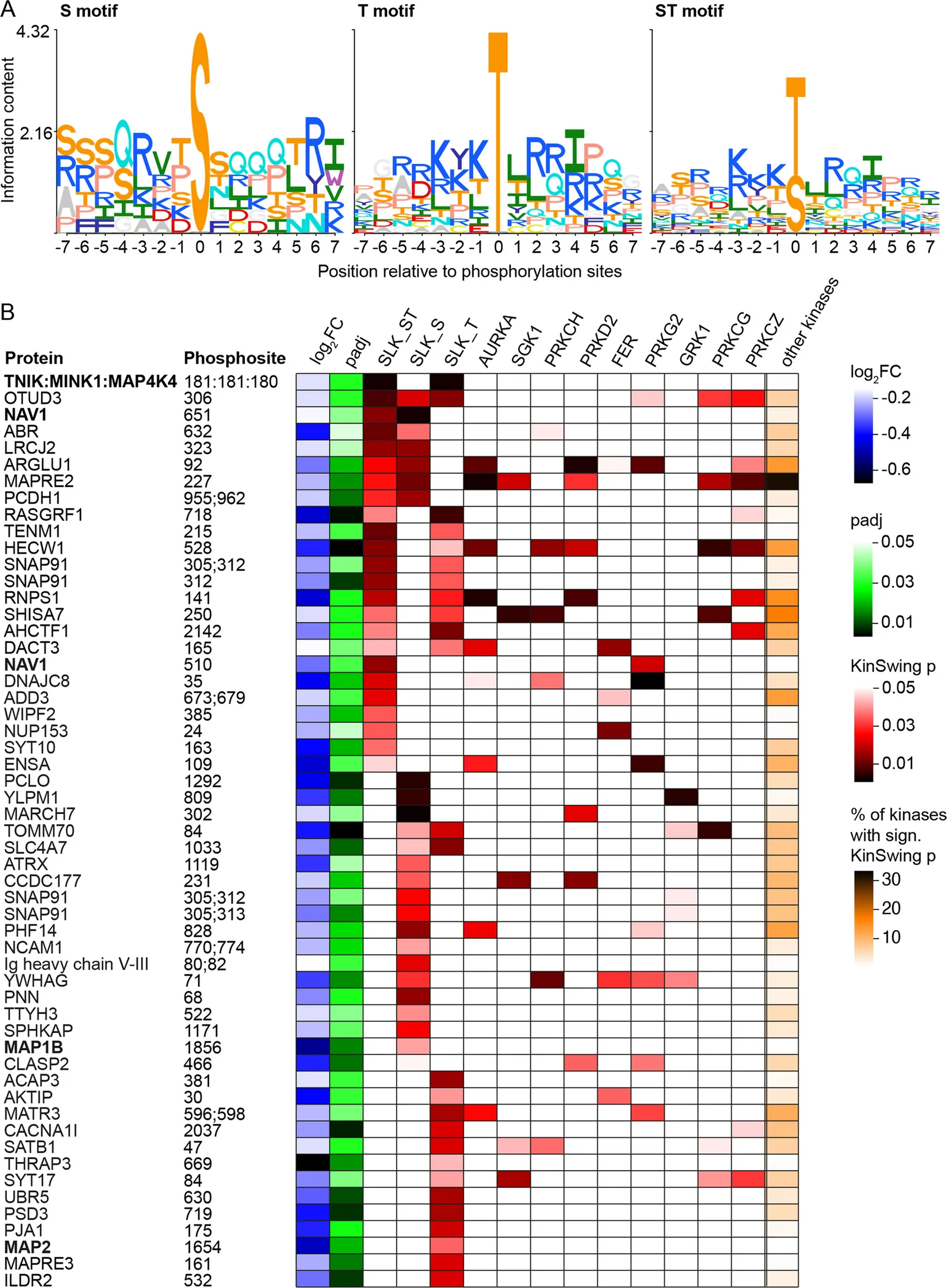
Identification of potential direct phosphorylation substrates of SLK. **A** In vitro phosphorylation motifs of SLK were computed based on known phosphorylation substrates. Separate motifs were modeled for serine (left) and threonine (middle) phosphorylation sites. A combined S/T motif (right) was created by merging the two. The motifs show distinct amino acid preferences surrounding the phosphosite. **B** Phosphorylation sites that were significantly less phosphorylated after SLK KD (padj < 0.05 and log_2_FC < 0) were analyzed with KinSwing to identify those matching the computed SLK motifs. All proteins with a phosphosite significantly matching at least one of the SLK motifs are shown. The phosphorylation sites were also compared to known motifs of 159 other kinases. The nine kinases whose predicted activity was significantly reduced after SLK KD are listed individually. For the remaining 150 kinases, the percentage of kinases with a significant KinSwing match to each phosphosite is indicated. The top candidates for each motif with a high specificity for SLK are marked in bold. If several proteins are listed in one row, the phosphorylated peptide is identical between them and cannot be assigned uniquely to one of these proteins

SLK KD resulted in increased phosphorylation of most KCNQ2 phosphorylation sites, indicating that these sites are indirectly regulated by signaling cascades downstream of SLK. To identify pathways that are directly regulated by SLK via substrate phosphorylation, we filtered the phosphoproteomics dataset for phosphorylation sites with reduced phosphorylation following SLK KD. However, decreased phosphorylation alone does not establish these proteins as direct substrates of SLK, as the observed changes may also result from altered activity of other kinases or phosphatases downstream of SLK. Therefore, we modeled substrate recognition motifs of SLK based on previously validated substrates (Fig. 5A, Table S1). As SLK is known to phosphorylate the amino acids serine and threonine, separate motifs were generated for serine, threonine, and the combined set of sites. Surprisingly, the motifs for serine and threonine phosphorylation differed substantially, with serine mainly being surrounded by polar, non-charged amino acids and threonine by basic amino acids.

Subsequently, we evaluated whether the phosphorylation sites identified in our mass spectrometry screen matched the predicted consensus sequence using the KinSwing algorithm [28]. In addition, the similarity of the respective phosphorylation site to computed and known motifs of 159 other kinases was assessed to analyze the probability of phosphorylation and motif specificity against other kinases. At the level of kinases, nine (AURKA, SGK1, PRKCH, PRKD2, FER, PRKG2, GRK1, PRKCG, PRKCZ) were predicted to be less active after SLK KD compared to control conditions (Fig. 5B), supporting a wider involvement of kinases upon SLK KD. When considering phosphorylation sites with reduced phosphorylation after SLK KD, 55 sites had a high probability of being phosphorylated by SLK based on the modeled SLK phosphorylation motifs. Identified proteins included cytoskeletal proteins (MAP1B, MAP2), cell adhesion molecules (NCAM1), and proteins with known functions at the synapse (MAPRE3, SHISA7) (Fig. 5B). While some phosphorylation sites (e.g. MAP1B_S1856, MAP2_S1654, and NAV1_S510) were predicted to be highly specific for SLK, others (e.g. in MAPRE2_S227) were predicted to be less specific, matching motifs of multiple kinases. Within the highly specific phosphorylation sites (predicted phosphorylation by less than 5 kinases), NAV1_S651 was the best hit for the modeled S motif and the kinases MINK1, TNIK, and MAP4K4 (T181:T181:T180) for the combined ST and T motif (Fig. 5B).

### The kinases MINK1 and TNIK are direct SLK substrates

**Fig. 6:**
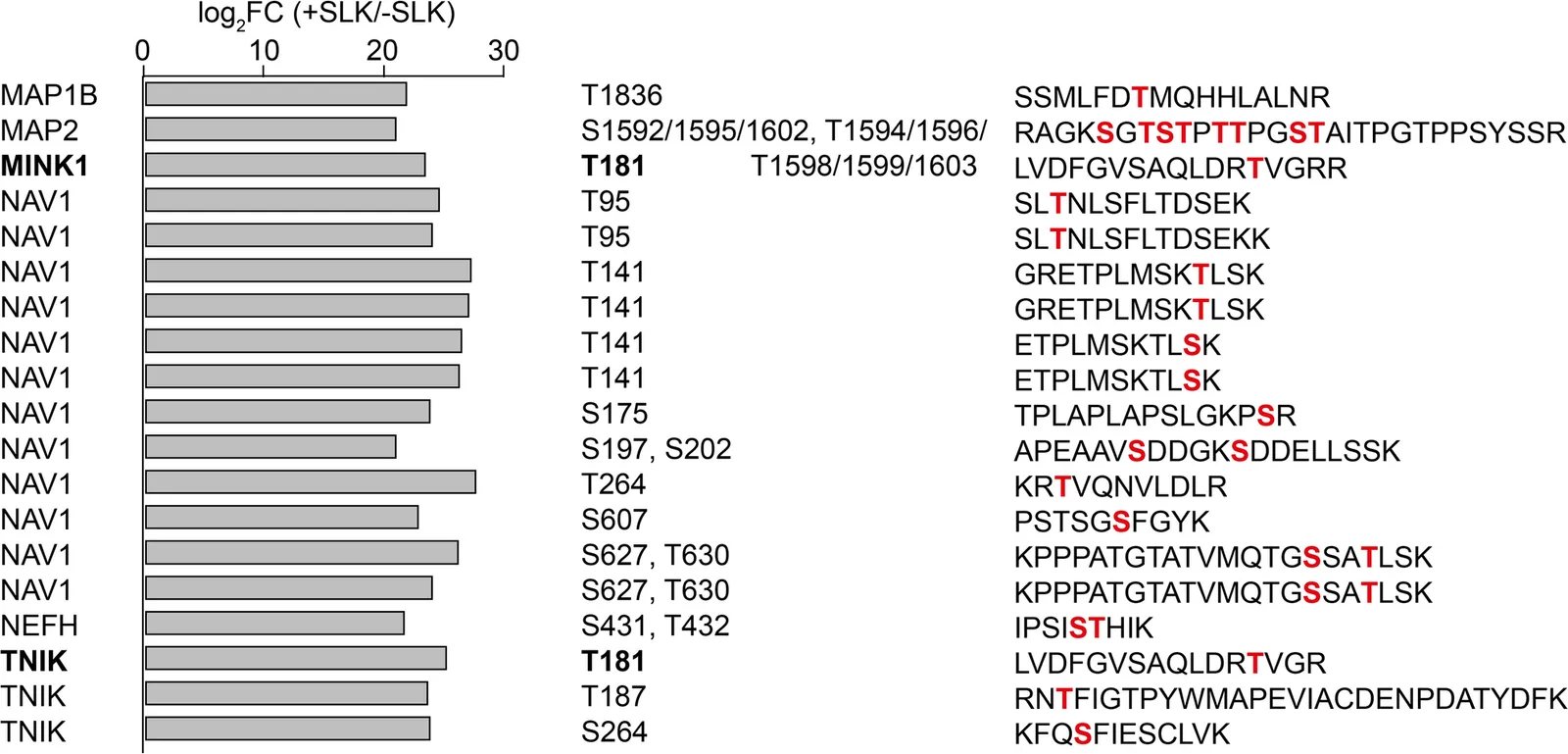
Verification of candidates identified in phosphoproteomic screen. Protein fragments including the phosphorylation site of interest were cloned with a GST-tag, purified, and subjected to in vitro kinase reactions with and without the kinase. For the depicted proteins, the phosphorylated peptide was only detected in the condition with SLK. If phosphorylation sites are listed more than once, different phosphopeptides or phosphopeptides with different modifications were detected. Detected peptides are listed on the right with the phosphorylation site marked in red. *n* = 1

To verify whether these proteins were direct SLK substrates, in vitro kinase reactions followed by mass spectrometric analysis were performed. Candidate proteins were selected based on the presence of phosphorylation sites matching the predicted SLK consensus motif (MAP1B, MAP2, MINK1, NAV1, TNIK) or their interaction with SLK combined with a strong reduction in phosphorylation upon SLK KD (CRMP1, MAP1B, NEFH, SV2A). Additionally, SHISA7 was tested as it had been validated to bind to SLK (Fig. 3D). Phosphorylation sites detected exclusively in kinase reactions with SLK, but not in kinase-free control reactions, were identified in MAP1B, MAP2, MINK1, NAV1, NEFH, and TNIK (Fig. 6).

**Fig. 7:**
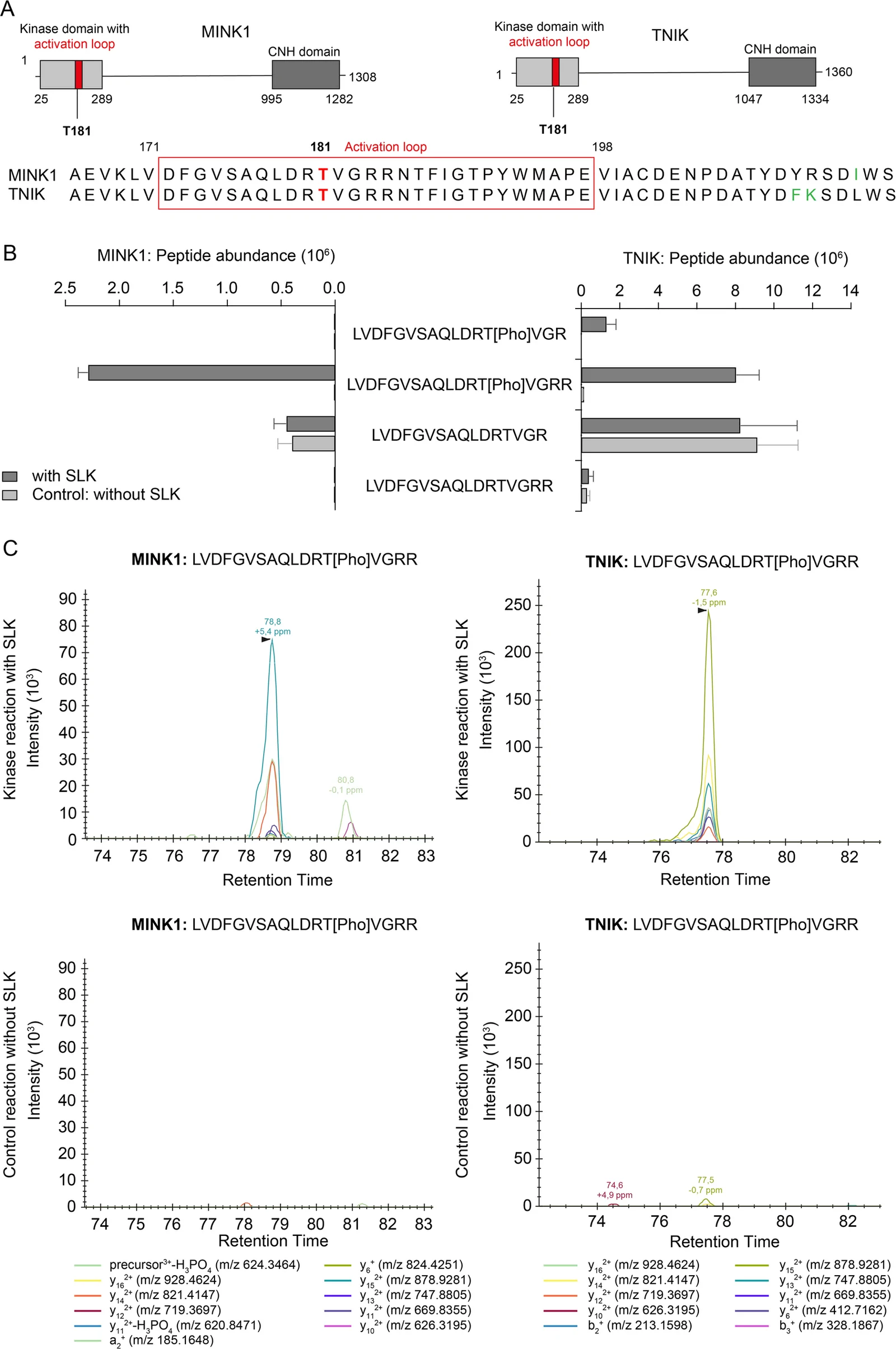
Phosphorylation of MINK1 and TNIK at T181 by SLK in vitro. **A** In kinase assays, SLK phosphorylated the kinases MINK1 and TNIK at threonine 181 (T181), which is located in the activation loop of their kinase domains. CNH: C-terminal citron homology domain. **B** The abundance of phosphorylated and non-phosphorylated peptides was quantified in reactions with SLK and in control reactions without SLK for MINK1 and TNIK. *n* = 4 independent assays. **C** Representative extracted fragment ion chromatograms are shown for the phosphorylated MINK1 and TNIK peptides in reactions with SLK and in control reactions without SLK

Two sites identified in the in vitro screen (MINK1_T181, TNIK_T181) corresponded to a phosphopeptide detected in the neuronal phosphoproteomics dataset. Targeted mass spectrometric analysis confirmed that both MINK1 and TNIK were specifically phosphorylated by SLK at T181, which is located in the activation loop of the kinase domains (Fig. 7A-C, Fig. S3A-C). Phosphorylated peptides were detected in the kinase reactions with SLK and not in the controls without SLK (Fig. 7B-C), validating that phosphorylation is mediated by SLK and not due to autophosphorylation. MINK1 and TNIK share 70% of their amino acid sequence, with the highest similarity in the C-terminal citron homology (CNH) and the N-terminal kinase domain where the phosphorylation site is located (Fig. S4). They are known to be involved in the outgrowth of dendrites, and a knockdown of either kinase resulted in a less complex dendritic tree [47]. These observations suggest that SLK may act upstream of MINK1 and TNIK signaling.

## Discussion

Our study maps two complementary layers of SLK signaling. The brain lysate-based AP-MS screen defines candidate SLK-binding proteins, whereas phosphoproteomics in cortical neurons identifies phosphorylation changes induced by SLK deficiency. Their intersection points to a role for SLK in synaptic and cytoskeletal signaling, and the biochemical assays identify MINK1 and TNIK as direct SLK substrates. Our data furthermore link SLK deficiency to altered neuronal network activity, altered KCNQ2 phosphorylation, and an increased sensitivity to KCNQ2 blockade. However, in contrast to MINK1 and TNIK, SLK appears to indirectly regulate phosphorylation sites in KCNQ2, most likely via kinase and phosphatase signaling networks.

AP-MS and phosphoproteomics were applied to identify different categories of proteins involved in SLK signaling: (1) SLK-binding proteins exhibiting SLK-dependent changes in their phosphorylation status. This group of proteins includes direct SLK substrates. (2) Proteins interacting with SLK but not showing a detectable phosphorylation change. Their function might be independent of kinase activity as kinases, including SLK, have been shown to exert kinase-independent functions [48]. For example, the non-catalytic C-terminal region of SLK is sufficient to promote actin stress-fiber disassembly, indicating that SLK can regulate cytoskeletal organization independently of its phosphotransferase activity [10, 49]. (3) Proteins whose phosphorylation status is directly or indirectly regulated by SLK through a phosphorylation cascade, but that are not physically associated with SLK. These proteins likely reflect indirect downstream signaling or transient kinase-substrate interactions that are not captured by AP-MS. Therefore, the AP-MS dataset provides new candidates for physical interaction networks, while the phosphoproteome dataset reveals SLK-dependent phosphorylation cascades. The integration of the two datasets enables the distinction between potential direct substrates and indirect downstream effectors, which can subsequently be validated. The two complementary datasets thereby provide a foundation for further studies to dissect SLK-dependent signaling pathways and determine their contribution to the processes regulated by SLK, including dendrite morphology, inhibitory synapse maturation, and neuronal network excitability.

Previous work showed that SLK controls the stabilization of inhibitory synapses and the branching of the dendritic tree of cortical neurons in the mouse brain [15, 16]. Consistent with this, our SLK interactome and phosphoproteomics screens identified a large number of dendritic, cytoskeletal, and synaptic proteins. Multiple proteins are established regulators of synaptic organization and neuronal signaling, including Neurexin-1 (NRXN1), Adhesion G protein-coupled receptor L3 (LPHN3), Regulating synaptic membrane exocytosis protein 1 (RIM1), Synapsin-1 (SYN1), Ras/Rap GTPase-activating protein SynGAP (SYNGAP1), Protein shisa-6 (SHISA6), Protein shisa-7 (SHISA7), and Synaptic vesicle glycoprotein 2A (SV2A), confirming that SLK is involved in the regulation of pre- and postsynaptic signaling [50–56]. SHISA7 could be validated as a direct interactor of SLK and exhibited reduced phosphorylation at S250 following SLK KD. SHISA7 is found at excitatory and inhibitory postsynaptic sites and interacts with AMPA and GABA_A_ receptor complexes [57–59]. Phosphorylation of SHISA7 regulates GABA_A_R trafficking at synaptic and extrasynaptic sites, and SHISA7 KD reduced the frequency but not the amplitude of mIPSCs, which mirrors the phenotype observed after SLK KD [15, 57, 58]. The presence of synaptic scaffold proteins such as Membrane-associated guanylate kinase, WW and PDZ domain-containing protein 3 (MAGI3), Disks large-associated protein 1 (DLGAP1), ARF GTPase-activating protein GIT1 (GIT1), and Tight junction protein 1 (TJP1) in our datasets further indicates that SLK may operate within large multiprotein signaling assemblies that coordinate receptor localization and signal integration at synapses [60–63].

The integrated dataset is also enriched for regulators of the neuronal cytoskeleton, such as Dihydropyrimidinase-related protein 1 (CRMP1), Microtubule-associated protein 1B (MAP1B), 2 (MAP2), and 4 (MAP4), Adducins (ADD1-3), Ankyrin-2 (ANK2) and 3 (ANK3), Band 4.1-like protein 1 (EPB41L1) and 3 (EPB41L3), Sorbin and SH3 domain-containing protein 1 (SORBS1), and Spectrin beta chain, non-erythrocytic 1 (SPNB2). CRMP1 was verified as a direct binding partner of SLK and showed a marked decrease in phosphorylation at S518 and S522 following SLK KD. S522 is known to be phosphorylated by Cyclin-dependent kinase 5 (CDK5) and subsequently at S518 by Glycogen synthase kinase-3 beta (GSK3B), which reduces its interaction with tubulin [64]. In addition, CRMP1 interacts with actin-binding proteins to regulate actin remodeling and filament elongation and fulfils a pronounced function in the growth cone during neurite outgrowth [64, 65]. As cytoskeletal remodeling is essential for the morphology of synapses and dendrites and for synaptic plasticity, modulation of these proteins could provide a mechanistic link between kinase activity and structural adaptations of neurons.

Several signaling molecules identified in the integrated dataset further support a role for SLK in coordinating intracellular signaling cascades. These include members of the Ras signaling pathway (RASGRF1, RASGRF2), Rho family guanine nucleotide exchange factors (FARP1, MCF2L, NGEF), kinases (MARK1, MINK1, MAST3, TNIK, PRKCG) and phosphatase regulatory proteins (PPP1R12A, PPP3CA, PPP6R2). The coexistence of multiple kinases and phosphatase components within the interaction network suggests that SLK functions as a broader phosphorylation-dependent signaling regulator to coordinate multiple signaling pathways involved in neuronal development.

One SLK-dependent phosphorylation signaling pathway identified in this study is the phosphorylation of the kinases MINK1 and TNIK by SLK. SLK is the first reported kinase to phosphorylate MINK1 and TNIK at T181 [66, 67]. The phosphorylation site is part of the activation loop of the kinase domains in TNIK and MINK1, and phosphorylation of T181 and T187 is known to be required for TNIK’s catalytic activation [68]. Since the kinase domain of MINK1 is nearly identical to that of TNIK, it is likely that phosphorylation of MINK1 at the homologous threonine similarly activates the kinase. MINK1 and TNIK belong to the family of GCK kinases, are enriched at postsynaptic sites, and are known to control dendritic morphology [47, 69–71]. They regulate actin dynamics and downstream pathways such as Hippo, MAP kinase, or JNK signaling, thereby modulating cytoskeletal remodeling, dendritic growth, and branching [67, 72–75]. Knockdown of either MINK1 or TNIK reduced dendritic arborization in cultured neurons, comparable to the phenotype observed after SLK KD [47, 76, 77]. These findings support a model in which SLK activates MINK1 and TNIK through phosphorylation, thereby propagating signals that contribute to the regulation of dendritic growth. This model will require further validation in future studies.

Several ion transporters and membrane-associated proteins (ATP1A3, ATP2B4, SLC12A2, SLC12A5, SLC4A4) also bound to SLK and exhibited altered phosphorylation after SLK KD, suggesting that SLK signaling may additionally regulate neuronal membrane excitability and ionic homeostasis. Together with the finding that SLK interacts with and regulates the phosphorylation of various synaptic proteins, this points to a broader role for SLK in modulating processes that shape neuronal communication and network function. In line with this, MEA recordings during synapse development and maturation revealed altered patterns of neuronal activity following SLK KD, with SLK KD neurons presenting with prolonged bursts and a higher number of spikes per burst. Longer bursts may suggest an impairment of mechanisms that normally terminate network activity. The ion channel protein with the highest number of altered phosphorylation sites after SLK KD was KCNQ2 (Kv7.2), a subunit of the M-type potassium channel that is crucial for controlling action potential firing and burst dynamics in neurons [78]. KCNQ2 was also identified in the AP-MS dataset as an SLK-associated protein. Application of the KCNQ2/Kv7.2 blocker ML252 to SLK-deficient neurons caused a strong reduction in the firing rate. ML252 also shortened the burst duration and decreased the number of spikes per burst in both control and SLK KD neurons, with a greater effect on SLK KD neurons. These findings indicate that SLK KD networks exhibit an enhanced sensitivity to pharmacological KCNQ2 blockade, but do not provide direct information about the M-current or KCNQ2 gating. The observed changes in neuronal network activity could be due to developmental alterations caused by SLK deficiency, for example, in network maturation or dendritic and synaptic morphology. However, as SLK KD induced a strong change in the phosphorylation pattern of KCNQ2, these changes could also be explained by alterations in the channel’s function triggered by post-translational modifications.

Post-translational modification of Kv7.2 channels can fine-tune neuronal excitability, and KCNQ2 function is regulated by phosphorylation via different protein kinases, such as protein kinase C (PKC) [79], extracellular signal-regulated kinase (ERK) [80], protein kinase A (PKA), CDK5, p38 mitogen-activated protein kinases (p38 MAPK), and GSK3B [81, 82]. In our screen, we detected eight differentially phosphorylated KCNQ2 sites (S52, S404, S418, S429, S448, S457, S483, and S681). PKA is known to phosphorylate KCNQ2 at S52 following cAMP stimulation, which increases M-currents [83]. S429, S448, and S457 are located within the Phosphatidylinositol 4,5-bisphosphate (PIP2)-binding domain of KCNQ2, and phosphorylation of this domain has been shown to modulate channel sensitivity to PIP2, thereby altering cellular excitability [82]. Together with the enhanced effect of KCNQ2 inhibition by ML252 in SLK KD neurons, the changed KCNQ2 phosphorylation pattern could suggest that SLK KD alters the neuromodulatory regulation of KCNQ2. Although the observed network phenotype cannot be attributed to individual KCNQ2 phosphosites, the phosphorylation of most of the differentially regulated sites is increased, suggesting that KCNQ2 may act downstream of one or more SLK-regulated kinase/phosphatase pathways. Identifying the upstream signaling components responsible for these site-specific changes will be important to determine how SLK signaling is coupled to KCNQ2 activity and how this contributes to the altered neuronal network phenotype.

Dysfunction of KCNQ2 is a well-established cause of early-onset epilepsy and developmental encephalopathy [84–86]. Studies that tested epilepsy-associated pathogenic KCNQ2 variants in neurons revealed a higher fraction of spikes in bursts and longer burst durations in MEA recordings [87, 88], which resembles the phenotype observed after SLK KD. In addition to KCNQ2, several other proteins identified in the SLK network are linked to neurodevelopmental disorders.

Cell adhesion molecules such as NRXN1 and scaffold proteins such as the SHANK proteins, DLGAP1, and CRMP1 were detected in the phosphoproteomics and AP-MS results and are implicated in ASD [60, 89–96]. Synaptic proteins such as GRIA1, CACNG2, PRRT2, and GRIN2A, which were differentially phosphorylated after SLK KD, are linked to ID and/or epilepsy [97–101]. The GTPase signaling proteins SYNGAP1, TRIO, and KALRN are known to be involved in ID, ASD, or developmental delay and were detected to be differentially phosphorylated after SLK KD [97, 102, 103]. Even cytoskeletal proteins such as MAP1B and MAP2, which were detected in both screens, are emerging as candidate genes for neurodevelopmental disorders, including ID, focal epilepsy, and developmental delay [104–107].

The two newly identified SLK substrates, MINK1 and TNIK, have been implicated in different human neurodevelopmental disorders, including epilepsy, autism spectrum disorder (ASD), and intellectual disability (ID) [74, 108–110]. Mice lacking TNIK exhibit synaptic abnormalities and cognitive deficits, recapitulating key features associated with human neurodevelopmental disorders [111]. A truncation variant of TNIK that results in complete protein loss was found in two families with ID [110]. The fact that SLK directly regulates MINK1 and TNIK suggests that SLK acts in the same neurodevelopmental pathways that, when disrupted, give rise to symptoms of the ID, ASD, and epilepsy spectra.

Emerging evidence also directly implicates SLK in neurodevelopmental disease. SLK expression is reduced in dysmorphic neurons of focal cortical dysplasias and gangliogliomas, which are developmental lesions associated with intractable epilepsy [15]. De novo mutations in the SLK gene have been identified in patients with various neurodevelopmental diseases, including ID and ASD [18, 19]. Recent data demonstrate that patient-derived SLK variants can result in loss of SLK kinase activity and impaired neuronal function [17]. These variants were linked to a recessive neurodevelopmental syndrome, providing direct evidence that SLK dysfunction can cause human neurodevelopmental pathology. This reinforces the view that SLK serves as a critical hub in neurodevelopmental signaling networks and that perturbation of SLK or its targets may converge on common developmental phenotypes.

Several limitations regarding the datasets need to be considered. The AP-MS experiment was designed as a discovery screen using immobilized SLK and whole-brain lysate of adult mice, rather than endogenous neuronal SLK complexes, and may include context-dependent interactions. Therefore, individual candidate proteins need to be validated in a neuronal context. The phosphoproteomics screen relied on one shRNA that was previously validated [15]; however, off-target contributions to individual phosphosites cannot be fully excluded. Nevertheless, the complementary datasets provide a valuable resource for future studies to identify and mechanistically dissect SLK-dependent signaling pathways.

Key next steps to further define the molecular mechanisms upstream and downstream of SLK include identifying the signals that control SLK activity in neurons and mapping the full complement of SLK substrates in vivo at different developmental stages. Such studies would deepen our understanding of how the Ste20-like kinase coordinates neuronal development and may reveal new therapeutic targets in neurodevelopmental disorders.

## Supplementary Information

Supplementary Material. 1

## Data Availability

The mass spectrometry proteomics data have been deposited to the ProteomeXchange Consortium with the dataset identifiers PXD072739 (Phosphoproteomics), PXD073661 (AP-MS and untargeted phosphorylation analysis), and PXD074031 (targeted phosphorylation analysis).

## Abbreviations

ACN: Acetonitrile
AP: Affinity purification
ASD: Autism spectrum disorder
BME: Basal Medium Eagle
CNH: Citron homology
DIV: Day in vitro
DMEM: Dulbecco’s Modified Eagle Medium
DTT: Dithiothreitol
E: Embryonic day
EDTA: Ethylenediaminetetraacetic acid
EGTA: Ethylene glycol-bis(β-aminoethyl ether)-N,N,N′,N′-tetraacetic acid
EThcD: Electron-transfer/higher-energy collision dissociation
FBS: Fetal Bovine Serum
GCK: Germinal center kinase
GO: Gene ontology
GST: Glutathione-S-transferase
HEK293T: Human Embryonic Kidney 293T
IB: Immunoblot
ID: Intellectual disability
IMDM: Iscove’s Modified Dulbecco’s Medium
IP: Immunoprecipitation
IPSC: Inhibitory postsynaptic current
IPTG: Isopropyl β-D-1-thiogalactopyranoside
KD: Knockdown
LC: Liquid chromatography
log_2_FC: Log_2_-fold change
MEA: Multi-electrode array
mIPSC: Miniature inhibitory postsynaptic current
MS: Mass spectrometry
padj: Adjusted p-value
PAGE: Polyacrylamide gel electrophoresis
PAK: P21-activated kinase
PCA: Principal component analysis
PIP2: Phosphatidylinositol 4,5-bisphosphate
PMSF: Phenylmethylsulfonyl fluoride
POI: Protein of interest
PWMs: Position weight matrices
shRNA: Short hairpin RNA
SLK: Ste20-like kinase

## References

[1] Humphrey SJ, James DE, Mann M. Protein phosphorylation: a major switch mechanism for metabolic regulation. Trends Endocrinol Metab. 2015;26:676–87. 10.1016/j.tem.2015.09.013 26498855

[2] Ardito F, Giuliani M, Perrone D, Troiano G, Lo ML. The crucial role of protein phosphorylation in cell signaling and its use as targeted therapy (Review). Int J Mol Med. 2017;40:271–80. 10.3892/ijmm.2017.3036 PMC5500920 28656226

[3] Nourbakhsh K, Yadav S. Kinase signaling in dendritic development and disease. Front Cell Neurosci. 2021;15:624648. 10.3389/fncel.2021.624648 PMC7902504 33642997

[4] Zhang K, Wang Y, Fan T, Zeng C, Sun ZS. The p21-activated kinases in neural cytoskeletal remodeling and related neurological disorders. Protein Cell. 2022;13:6–25. 10.1007/s13238-020-00812-9 PMC8776968 33306168

[5] Kyriakis JM. Signaling by the germinal center kinase family of protein kinases. J Biol Chem. 1999;274:5259–62. 10.1074/jbc.274.9.5259 10026130

[6] Dan I, Watanabe NM, Kusumi A. The Ste20 group kinases as regulators of MAP kinase cascades. Trends Cell Biol. 2001;11:220–30. 10.1016/s0962-8924(01)01980-8 11316611

[7] Ultanir SK, Yadav S, Hertz NT, Oses-Prieto JA, Claxton S, Burlingame AL, et al. MST3 kinase phosphorylates TAO1/2 to enable Myosin Va function in promoting spine synapse development. Neuron. 2014;84:968–82. 10.1016/j.neuron.2014.10.025 PMC4407996 25456499

[8] Byeon S, Yadav S. Pleiotropic functions of TAO kinases and their dysregulation in neurological disorders. Sci Signal. 2024;17:eadg0876. 10.1126/scisignal.adg0876 PMC11810052 38166033

[9] Yadav S, Oses-Prieto JA, Peters CJ, Zhou J, Pleasure SJ, Burlingame AL, et al. TAOK2 kinase mediates PSD95 stability and dendritic spine maturation through Septin7 phosphorylation. Neuron. 2017;93:379–93. 10.1016/j.neuron.2016.12.006 PMC5267388 28065648

[10] Sabourin L, Tamai K, Seale P, Wagner J, Rudnicki M. Caspase 3 cleavage of the Ste20-related kinase SLK releases and activates an apoptosis-inducing kinase domain and an actin-disassembling region. Mol Cell Biol. 2000;20:684–96. 10.1128/mcb.20.2.684-696.2000 PMC85169 10611247

[11] Wagner S, Flood TA, O’Reilly P, Hume K, Sabourin LA. Association of the Ste20-like kinase (SLK) with the microtubule. Role in Rac1-mediated regulation of actin dynamics during cell adhesion and spreading. J Biol Chem. 2002;277:37685–92. 10.1074/jbc.M205899200 12151406

[12] Wagner S, Storbeck CJ, Roovers K, Chaar ZY, Kolodziej P, McKay M, et al. FAK/src-family dependent activation of the Ste20-like kinase SLK is required for microtubule-dependent focal adhesion turnover and cell migration. PLoS ONE. 2008;3:e1868. 10.1371/journal.pone.0001868 PMC2270904 18382658

[13] O’Reilly PG, Wagner S, Franks DJ, Cailliau K, Browaeys E, Dissous C, et al. The Ste20-like kinase SLK is required for cell cycle progression through G2. J Biol Chem. 2005;280:42383–90. 10.1074/jbc.M510763200 16236704

[14] Delarosa S, Guillemette J, Papillon J, Han YS, Kristof AS, Cybulsky AV. Activity of the Ste20-like kinase, SLK, is enhanced by homodimerization. Am J Physiol Renal Physiol. 2011;301:F554–64. 10.1152/ajprenal.00062.2011 PMC3174548 21677149

[15] Schoch S, Quatraccioni A, Robens BK, et al. Ste20-like kinase is critical for inhibitory synapse maintenance and its deficiency confers a developmental dendritopathy. J Neurosci. 2021;41:8111–25. 10.1523/JNEUROSCI.0352-21.2021 PMC8482859 34400520

[16] Royero P, Quatraccioni A, Früngel R, et al. Circuit-selective cell-autonomous regulation of inhibition in pyramidal neurons by Ste20-like kinase. Cell Rep. 2022;41:111757. 10.1016/j.celrep.2022.111757 PMC9756112 36476865

[17] Alabdi L, Altuwaijri N, Zhu J, et al. SLK is mutated in individuals with a neurodevelopmental disorder. EBioMedicine. 2025;116:105725. 10.1016/j.ebiom.2025.105725 PMC12139437 40347834

[18] Jiang YH, Yuen RKC, Jin X, et al. Detection of clinically relevant genetic variants in autism spectrum disorder by whole-genome sequencing. Am J Hum Genet. 2013;93:249–63. 10.1016/j.ajhg.2013.06.012 PMC3738824 23849776

[19] Anazi S, Maddirevula S, Salpietro V, et al. Expanding the genetic heterogeneity of intellectual disability. Hum Genet. 2017;136:1419–29. 10.1007/s00439-017-1843-2 28940097

[20] Van Loo KMJ, Schaub C, Pitsch J, et al. Zinc regulates a key transcriptional pathway for epileptogenesis via metal-regulatory transcription factor 1. Nat Commun. 2015;6:8688. 10.1038/ncomms9688 PMC4846312 26498180

[21] Gleichman AJ, Spruce LA, Dalmau J, Seeholzer SH, Lynch DR. Anti-NMDA receptor encephalitis antibody binding is dependent on amino acid identity of a small region within the GluN1 amino terminal domain. J Neurosci. 2012;32:11082–94. 10.1523/JNEUROSCI.0064-12.2012 PMC3430387 22875940

[22] Müller JA, Betzin J, Santos-Tejedor J, et al. A presynaptic phosphosignaling hub for lasting homeostatic plasticity. Cell Rep. 2022;39:110696. 10.1016/j.celrep.2022.110696 35443170

[23] Rosenfeld J, Capdevielle J, Guillemot JC, Ferrara P. In-gel digestion of proteins for internal sequence analysis after one- or two-dimensional gel electrophoresis. Anal Biochem. 1992;203:173–9. 10.1016/0003-2697(92)90061-b 1524213

[24] Jenö P, Mini T, Moes S, Hintermann E, Horst M. Internal sequences from proteins digested in polyacrylamide gels. Anal Biochem. 1995;224:75–82. 10.1006/abio.1995.1010 7710119

[25] Käll L, Storey JD, MacCoss MJ, Noble WS. Assigning significance to peptides identified by tandem mass spectrometry using decoy databases. J Proteome Res. 2008;7:29–34. 10.1021/pr700600n 18067246

[26] Winter D, Seidler J, Ziv Y, Shiloh Y, Lehmann WD. Citrate boosts the performance of phosphopeptide analysis by UPLC-ESI-MS/MS. J Proteome Res. 2009;8:418–24. 10.1021/pr800304n 19053530

[27] Pino LK, Searle BC, Bollinger JG, Nunn B, MacLean B, MacCoss MJ. The Skyline ecosystem: informatics for quantitative mass spectrometry proteomics. Mass Spectrom Rev. 2020;39:229–44. 10.1002/mas.21540 PMC5799042 28691345

[28] Engholm-Keller K, Waardenberg AJ, Müller JA, Wark JR, Fernando RN, Arthur JW, et al. The temporal profile of activity-dependent presynaptic phospho-signalling reveals long-lasting patterns of poststimulus regulation. PLoS Biol. 2019;17:e3000170. 10.1371/journal.pbio.3000170 PMC6415872 30822303

[29] Dudoit S, Fridlyand J. A prediction-based resampling method for estimating the number of clusters in a dataset. Genome Biol. 2002;3:RESEARCH0036. 10.1186/gb-2002-3-7-research0036 PMC126241 12184810

[30] Leek JT, Storey JD. Capturing heterogeneity in gene expression studies by surrogate variable analysis. PLoS Genet. 2007;3:1724–35. 10.1371/journal.pgen.0030161 PMC1994707 17907809

[31] Ritchie ME, Phipson B, Wu D, Hu Y, Law CW, Shi W, et al. Limma powers differential expression analyses for RNA-sequencing and microarray studies. Nucleic Acids Res. 2015;43:e47. 10.1093/nar/gkv007 PMC4402510 25605792

[32] Benjamini Y, Hochberg Y. Controlling the false discovery rate: a practical and powerful approach to multiple testing. J Roy Stat Soc: Ser B (Methodol). 1995;57:289–300.

[33] Ballman KV, Grill DE, Oberg AL, Therneau TM. Faster cyclic loess: normalizing RNA arrays via linear models. Bioinformatics. 2004;20:2778–86. 10.1093/bioinformatics/bth327 15166021

[34] Sherman BT, Hao M, Qiu J, Jiao X, Baseler MW, Lane HC, et al. DAVID: a web server for functional enrichment analysis and functional annotation of gene lists (2021 update). Nucleic Acids Res. 2022;50:W216–21. 10.1093/nar/gkac194 PMC9252805 35325185

[35] Huang DW, Sherman BT, Lempicki RA. Systematic and integrative analysis of large gene lists using DAVID bioinformatics resources. Nat Protoc. 2009;4:44–57. 10.1038/nprot.2008.211 19131956

[36] Koopmans F, van Nierop P, Andres-Alonso M, et al. SynGO: an evidence-based, expert-curated knowledge base for the synapse. Neuron. 2019;103:217-234.e4. 10.1016/j.neuron.2019.05.002 PMC6764089 31171447

[37] Szklarczyk D, Nastou K, Koutrouli M, et al. The STRING database in 2025: protein networks with directionality of regulation. Nucleic Acids Res. 2025;53:D730–7. 10.1093/nar/gkae1113 PMC11701646 39558183

[38] Sharma K, Schmitt S, Bergner CG, et al. Cell type- and brain region-resolved mouse brain proteome. Nat Neurosci. 2015;18:1819–31. 10.1038/nn.4160 PMC7116867 26523646

[39] Franz M, Lopes CT, Fong D, Kucera M, Cheung M, Siper MC, et al. Cytoscape.js 2023 update: a graph theory library for visualization and analysis. Bioinformatics. 2023;39:btad031. 10.1093/bioinformatics/btad031 PMC9889963 36645249

[40] Kolberg L, Raudvere U, Kuzmin I, Adler P, Vilo J, Peterson H. g:Profiler-interoperable web service for functional enrichment analysis and gene identifier mapping (2023 update). Nucleic Acids Res. 2023;51:W207–12. 10.1093/nar/gkad347 PMC10320099 37144459

[41] Takai Y, Sasaki T, Matozaki T. Small GTP-binding proteins. Physiol Rev. 2001;81:153–208. 10.1152/physrev.2001.81.1.153 11152757

[42] Wilkinson B, Coba MP. Molecular architecture of postsynaptic interactomes. Cell Signal. 2020;76:109782. 10.1016/j.cellsig.2020.109782 PMC7669586 32941943

[43] Kaizuka T, Takumi T. Alteration of synaptic protein composition during developmental synapse maturation. Eur J Neurosci. 2024;59:2894–914. 10.1111/ejn.16304 38571321

[44] Coba MP. Regulatory mechanisms in postsynaptic phosphorylation networks. Curr Opin Struct Biol. 2019;54:86–94. 10.1016/j.sbi.2019.01.003 PMC7018365 30807903

[45] Sheng M, Kim E. The postsynaptic organization of synapses. Cold Spring Harb Perspect Biol. 2011;3:a005678. 10.1101/cshperspect.a005678 PMC3225953 22046028

[46] Mozhayeva MG, Sara Y, Liu X, Kavalali ET. Development of vesicle pools during maturation of hippocampal synapses. J Neurosci. 2002;22:654–65. 10.1523/JNEUROSCI.22-03-00654.2002 PMC6758530 11826095

[47] Hussain NK, Hsin H, Huganir RL, Sheng M. MINK and TNIK differentially act on Rap2-mediated signal transduction to regulate neuronal structure and AMPA receptor function. J Neurosci. 2010;30:14786–94. 10.1523/JNEUROSCI.4124-10.2010 PMC3070943 21048137

[48] Rauch J, Volinsky N, Romano D, Kolch W. The secret life of kinases: functions beyond catalysis. Cell Commun Signal. 2011;9:23. 10.1186/1478-811X-9-23 PMC3215182 22035226

[49] Panneton V, Nath A, Sader F, Delaunay N, Pelletier A, Maier D, et al. Regulation of catalytic and non-catalytic functions of the *Drosophila* Ste20 kinase Slik by activation segment phosphorylation. J Biol Chem. 2015;290:20960–71. 10.1074/jbc.M115.645952 PMC4543655 26170449

[50] Rossi R, Arjmand S, Bærentzen SL, Gjedde A, Landau AM. Synaptic vesicle glycoprotein 2A: features and functions. Front Neurosci. 2022;16:864514. 10.3389/fnins.2022.864514 PMC9096842 35573314

[51] Abdollahi Nejat M, Klaassen RV, Spijker S, Guus Smit AB. Auxiliary subunits of the AMPA receptor: the Shisa family of proteins. Curr Opin Pharmacol. 2021;58:52–61. 10.1016/j.coph.2021.03.001 33892364

[52] Llamosas N, Arora V, Vij R, et al. SYNGAP1 controls the maturation of dendrites, synaptic function, and network activity in developing human neurons. J Neurosci. 2020;40:7980–94. 10.1523/JNEUROSCI.1367-20.2020 PMC7548701 32887745

[53] Perlini LE, Botti F, Fornasiero EF, Giannandrea M, Bonanomi D, Amendola M, et al. Effects of phosphorylation and neuronal activity on the control of synapse formation by synapsin I. J Cell Sci. 2011;124:3643–53. 10.1242/jcs.086223 22045728

[54] Kaeser PS, Deng L, Fan M, Südhof TC. RIM genes differentially contribute to organizing presynaptic release sites. Proc Natl Acad Sci U S A. 2012;109:11830–5. 10.1073/pnas.1209318109 PMC3406861 22753485

[55] O’Sullivan ML, Martini F, von Daake S, Comoletti D, Ghosh A. LPHN3, a presynaptic adhesion-GPCR implicated in ADHD, regulates the strength of neocortical layer 2/3 synaptic input to layer 5. Neural Dev. 2014;9:7. 10.1186/1749-8104-9-7 PMC3996519 24739570

[56] Trotter JH, Hao J, Maxeiner S, Tsetsenis T, Liu Z, Zhuang X, et al. Synaptic neurexin-1 assembles into dynamically regulated active zone nanoclusters. J Cell Biol. 2019;218:2677–98. 10.1083/jcb.201812076 PMC6683742 31262725

[57] Wu K, Shepard RD, Castellano D, Han W, Tian Q, Dong L, et al. Shisa7 phosphorylation regulates GABAergic transmission and neurodevelopmental behaviors. Neuropsychopharmacology. 2022;47:2160–70. 10.1038/s41386-022-01334-0 PMC9556544 35534528

[58] Han W, Li J, Pelkey KA, et al. Shisa7 is a GABAA receptor auxiliary subunit controlling benzodiazepine actions. Science. 2019;366:246–50. 10.1126/science.aax5719 PMC7382361 31601770

[59] Schmitz LJM, Klaassen RV, Ruiperez-Alonso M, et al. The AMPA receptor-associated protein Shisa7 regulates hippocampal synaptic function and contextual memory. Elife. 2017;6:e24192. 10.7554/eLife.24192 PMC5737659 29199957

[60] Rasmussen AH, Rasmussen HB, Silahtaroglu A. The DLGAP family: neuronal expression, function and role in brain disorders. Mol Brain. 2017;10:43. 10.1186/s13041-017-0324-9 PMC5583998 28870203

[61] Smith KR, Davenport EC, Wei J, Li X, Pathania M, Vaccaro V, et al. GIT1 and βPIX are essential for GABA(A) receptor synaptic stability and inhibitory neurotransmission. Cell Rep. 2014;9:298–310. 10.1016/j.celrep.2014.08.061 PMC4536293 25284783

[62] Marsh AJ, Michel JC, Adke AP, Heckman EL, Miller AC. Asymmetry of an intracellular scaffold at vertebrate electrical synapses. Curr Biol. 2017;27:3561. 10.1016/j.cub.2017.10.011 PMC5698123 29103941

[63] Zheng CY, Seabold GK, Horak M, Petralia RS. MAGUKs, synaptic development, and synaptic plasticity. Neuroscientist. 2011;17:493–512. 10.1177/1073858410386384 PMC3191319 21498811

[64] Nakamura F, Ohshima T, Goshima Y. Collapsin response mediator proteins: their biological functions and pathophysiology in neuronal development and regeneration. Front Cell Neurosci. 2020;14:188. 10.3389/fncel.2020.00188 PMC7325199 32655376

[65] Higurashi M, Iketani M, Takei K, Yamashita N, Aoki R, Kawahara N, et al. Localized role of CRMP1 and CRMP2 in neurite outgrowth and growth cone steering. Dev Neurobiol. 2012;72:1528–40. 10.1002/dneu.22017 22378692

[66] Sheela A, Subair S, Ummar S, Mahin A, Gopalakrishnan AP, Raju R, et al. A system-wide investigation into the phosphoregulatory network of TNIK and its cellular implications. Front Bioinform. 2026;6:1722876. 10.3389/fbinf.2026.1722876 PMC13021626 41909809

[67] Kot A, Koszewska D, Ochman B, Świętochowska E. Clinical potential of misshapen/NIKs-related kinase (MINK) 1-a many-sided element of cell physiology and pathology. Curr Issues Mol Biol. 2024;46:13811–45. 10.3390/cimb46120826 PMC11727420 39727954

[68] Wang Q, Amato SP, Rubitski DM, Hayward MH, Kormos BL, Verhoest PR, et al. Identification of phosphorylation consensus sequences and endogenous neuronal substrates of the psychiatric risk kinase TNIK. J Pharmacol Exp Ther. 2016;356:410–23. 10.1124/jpet.115.229880 26645429

[69] Wang Q, Charych EI, Pulito VL, et al. The psychiatric disease risk factors DISC1 and TNIK interact to regulate synapse composition and function. Mol Psychiatry. 2011;16:1006–23. 10.1038/mp.2010.87 PMC3176992 20838393

[70] Burette AC, Phend KD, Burette S, et al. Organization of TNIK in dendritic spines. J Comp Neurol. 2015;523:1913. 10.1002/cne.23770 PMC4509985 25753355

[71] Baltussen LL, Rosianu F, Ultanir SK. Kinases in synaptic development and neurological diseases. Prog Neuropsychopharmacol Biol Psychiatry. 2018;84:343–52. 10.1016/j.pnpbp.2017.12.006 29241837

[72] Meng Z, Moroishi T, Mottier-Pavie V, et al. MAP4K family kinases act in parallel to MST1/2 to activate LATS1/2 in the Hippo pathway. Nat Commun. 2015;5:8357. 10.1038/ncomms9357 PMC4600732 26437443

[73] Larhammar M, Huntwork-Rodriguez S, Rudhard Y, Sengupta-Ghosh A, Lewcock JW. The Ste20 family kinases MAP4K4, MINK1, and TNIK converge to regulate stress-induced JNK signaling in neurons. J Neurosci. 2017;37:11074–84. 10.1523/JNEUROSCI.0905-17.2017 PMC6596808 28993483

[74] Jiang J, Wilkinson B, Flores I, et al. Mutations in the postsynaptic density signaling hub TNIK disrupt PSD signaling in human models of neurodevelopmental disorders. Front Mol Neurosci. 2024;17:1359154. 10.3389/fnmol.2024.1359154 PMC11024424 38638602

[75] Taira K, Umikawa M, Takei K, Myagmar BE, Shinzato M, Machida N, et al. The Traf2- and Nck-interacting kinase as a putative effector of Rap2 to regulate actin cytoskeleton. J Biol Chem. 2004;279:49488–96. 10.1074/jbc.M406370200 15342639

[76] Kawabe H, Neeb A, Dimova K, et al. Regulation of Rap2A by the ubiquitin ligase Nedd4-1 controls neurite development. Neuron. 2010;65:358–72. 10.1016/j.neuron.2010.01.007 PMC2825371 20159449

[77] Lasham DJ, Arta RK, Hadi AF, Egawa J, Lemmon VP, Takasugi T, et al. Effects of MAP4K inhibition on neurite outgrowth. Mol Brain. 2023;16:79. 10.1186/s13041-023-01066-2 PMC10656890 37980537

[78] Brown DA, Passmore GM. Neural KCNQ (Kv7) channels. Br J Pharmacol. 2009;156:1185–95. 10.1111/j.1476-5381.2009.00111.x PMC2697739 19298256

[79] Kosenko A, Kang S, Smith IM, Greene DL, Langeberg LK, Scott JD, et al. Coordinated signal integration at the M-type potassium channel upon muscarinic stimulation. EMBO J. 2012;31:3147–56. 10.1038/emboj.2012.156 PMC3400014 22643219

[80] Tsuboi D, Otsuka T, Shimomura T, et al. Dopamine drives neuronal excitability via KCNQ channel phosphorylation for reward behavior. Cell Rep. 2022;40:111309. 10.1016/j.celrep.2022.111309 36070693

[81] Erdem FA, Salzer I, Heo S, Chen WQ, Jung G, Lubec G, et al. Updating in vivo and in vitro phosphorylation and methylation sites of voltage-gated Kv7.2 potassium channels. Proteomics. 2017;17:10.1002. 10.1002/pmic.201700015 PMC6349142 28834300

[82] Salzer I, Erdem FA, Chen WQ, Heo S, Koenig X, Schicker KW, et al. Phosphorylation regulates the sensitivity of voltage-gated Kv7.2 channels towards phosphatidylinositol-4,5-bisphosphate. J Physiol. 2017;595:759–76. 10.1113/JP273274 PMC5215842 27621207

[83] Schroeder BC, Kubisch C, Stein V, Jentsch TJ. Moderate loss of function of cyclic-AMP-modulated KCNQ2/KCNQ3 K+ channels causes epilepsy. Nature. 1998;396:687–90. 10.1038/25367 9872318

[84] Orhan G, Bock M, Schepers D, et al. Dominant-negative effects of KCNQ2 mutations are associated with epileptic encephalopathy. Ann Neurol. 2014;75:382–94. 10.1002/ana.24080 24318194

[85] Miceli F, Soldovieri MV, Weckhuysen S, Cooper E, Taglialatela M. KCNQ2-Related Disorders. GeneReviews: University of Washington, Seattle (WA); 2010. 20437616

[86] Chokvithaya S, Caengprasath N, Buasong A, et al. Nine patients with KCNQ2-related neonatal seizures and functional studies of two missense variants. Sci Rep. 2023;13:3328. 10.1038/s41598-023-29924-y PMC9971330 36849527

[87] Simkin D, Wafa SMA, Gharib M, Marshall KA, Yang Y, Laux LC et al. Machine learning resolves functional phenotypes and therapeutic responses in KCNQ2 developmental epileptic encephalopathy iPSC models. bioRxiv (Preprint). 2025; 2025.07.26.666977.

[88] Sundberg M, Shum C, Norabuena EM, et al. Human iPSC-derived glutamatergic neurons with pathogenic KCNQ2 variants display hyperactive bursting phenotypes. Neurobiol Dis. 2025;216:107126. 10.1016/j.nbd.2025.107126 PMC12745820 41015095

[89] Liu T, Shi Y, Chan MTV, et al. Developmental protein kinase C hyper-activation results in microcephaly and behavioral abnormalities in zebrafish. Transl Psychiatry. 2018;8:232. 10.1038/s41398-018-0285-5 PMC6199330 30352990

[90] Montalbano S, Krebs MD, Rosengren A, et al. Analysis of exonic deletions in a large population study provides novel insights into NRXN1 pathology. NPJ Genom Med. 2024;9:67. 10.1038/s41525-024-00450-8 PMC11655628 39695155

[91] Onay H, Kacamak D, Kavasoglu AN, Akgun B, Yalcinli M, Kose S, et al. Mutation analysis of the NRXN1 gene in autism spectrum disorders. Balkan J Med Genet. 2017;19:17–22. 10.1515/bjmg-2016-0031 PMC5343326 28289584

[92] Silver H, Greenberg R, Siper PM, et al. Protein-truncating variants and deletions of SHANK2 are associated with autism spectrum disorder and other neurodevelopmental concerns. J Neurodev Disord. 2025;17:25. 10.1186/s11689-025-09600-0 PMC12042525 40307697

[93] Caumes R, Smol T, Thuillier C, Balerdi M, Lestienne-Roche C, Manouvrier-Hanu S, et al. Phenotypic spectrum of SHANK2-related neurodevelopmental disorder. Eur J Med Genet. 2020;63:104072. 10.1016/j.ejmg.2020.104072 32987185

[94] Ravindran E, Arashiki N, Becker LL, et al. Monoallelic CRMP1 gene variants cause neurodevelopmental disorder. Elife. 2022;11:e80793. 10.7554/eLife.80793 PMC9803352 36511780

[95] Liu J, Wang Q, Chen J. Case report: a de novo variant of CRMP1 in an individual with a neurodevelopmental disorder. Front Neurosci. 2024;18 :1490731. 10.3389/fnins.2024.1490731 PMC11695366 39758889

[96] Monteiro P, Feng G. SHANK proteins: roles at the synapse and in autism spectrum disorder. Nat Rev Neurosci. 2017;18:147–57. 10.1038/nrn.2016.183 28179641

[97] Forrest MP, Parnell E, Penzes P. Dendritic structural plasticity and neuropsychiatric disease. Nat Rev Neurosci. 2018;19:215–34. 10.1038/nrn.2018.16 PMC6442683 29545546

[98] Strehlow V, Myers KA, Morgan AT, Scheffer IE, Lemke JR. GRIN2A-Related Disorders. GeneReviews: University of Washington, Seattle (WA); 2024. 27683935

[99] Tvergaard NK, Tkemaladze T, Stödberg T, Kvarnung M, Tatton-Brown K, Baralle D, et al. Unraveling GRIA1 neurodevelopmental disorders: lessons learned from the p. (Ala636Thr) variant. Clin Genet. 2024;106:427–36. 10.1111/cge.14577 38890806

[100] Hamdan FF, Gauthier J, Araki Y, et al. Excess of de novo deleterious mutations in genes associated with glutamatergic systems in nonsyndromic intellectual disability. Am J Hum Genet. 2011;88:306–16. 10.1016/j.ajhg.2011.02.001 PMC3059427 21376300

[101] Yang K, Quiroz V, Ebrahimi-Fakhari D. PRRT2-Related Disorder. GeneReviews: University of Washington, Seattle (WA); 2024. 29334453

[102] Bednarczuk N, Housby H, Lee IO, Consortium IMAGINE, Skuse D, Wolstencroft J. Behavioural and neurodevelopmental characteristics of SYNGAP1. J Neurodev Disord. 2024;16:46. 10.1186/s11689-024-09563-8 PMC11325819 39148034

[103] Varvagiannis K, Vissers LE, Baralle D, de Vries BB, Gazdagh G. TRIO-Related Neurodevelopmental Disorder. GeneReviews: University of Washington, Seattle (WA); 2023. 28796471

[104] Walters GB, Gustafsson O, Sveinbjornsson G, et al. MAP1B mutations cause intellectual disability and extensive white matter deficit. Nat Commun. 2018;9:3456. 10.1038/s41467-018-05595-6 PMC6110722 30150678

[105] Julca DM, Diaz J, Berger S, Leon E. MAP1B related syndrome: case presentation and review of literature. Am J Med Genet A. 2019;179:1703–8. 10.1002/ajmg.a.61280 31317654

[106] Archer J, Edwards M, Macdougall T, Baxter A, Goel H. MAP1B variants disrupt neuronal migration: insights from three novel families. Clin Genet. 2026;109:558–63. 10.1111/cge.70059 PMC12881218 40874586

[107] Westphal DS, Andres S, Makowski C, Meitinger T, Hoefele J. MAP2 - a candidate gene for epilepsy, developmental delay and behavioral abnormalities in a patient with microdeletion 2q34. Front Genet. 2018;9:99. 10.3389/fgene.2018.00099 PMC5879085 29632546

[108] Wang M, Gu Y, Li Q, Feng B, Lv X, Zhang H, et al. The Traf2 and NcK interacting kinase inhibitor NCB-0846 suppresses seizure activity involving the decrease of GRIA1. Genes Dis. 2023;11:100997. 10.1016/j.gendis.2023.03.036 PMC10826163 38292191

[109] Eisfeldt J, Schuy J, Stattin EL, Kvarnung M, Falk A, Feuk L, et al. Multi-omic investigations of a 17-19 translocation links MINK1 disruption to autism, epilepsy and osteoporosis. Int J Mol Sci. 2022;23:9392. 10.3390/ijms23169392 PMC9408972 36012658

[110] Anazi S, Shamseldin HE, AlNaqeb D, Abouelhoda M, Monies D, Salih MA, et al. A null mutation in TNIK defines a novel locus for intellectual disability. Hum Genet. 2016;135:773–8. 10.1007/s00439-016-1671-9 27106596

[111] Coba MP, Komiyama NH, Nithianantharajah J, et al. TNiK is required for postsynaptic and nuclear signaling pathways and cognitive function. J Neurosci. 2012;32:13987–99. 10.1523/JNEUROSCI.2433-12.2012 PMC3978779 23035106

