# Supplementary material for: Identification of a Ste20-like kinase-dependent signaling network orchestrating cytoskeletal and synaptic development of cortical neurons
Source: Cell Commun Signal. 2026 Oct 2;24:528. doi: 10.1186/s12964-026-03250-y (PMC13632193; doi:10.1186/s12964-026-03250-y)
Supplement: Supplementary file 1 [file 12964_2026_3250_MOESM1_ESM.pdf]

Supplementary Files

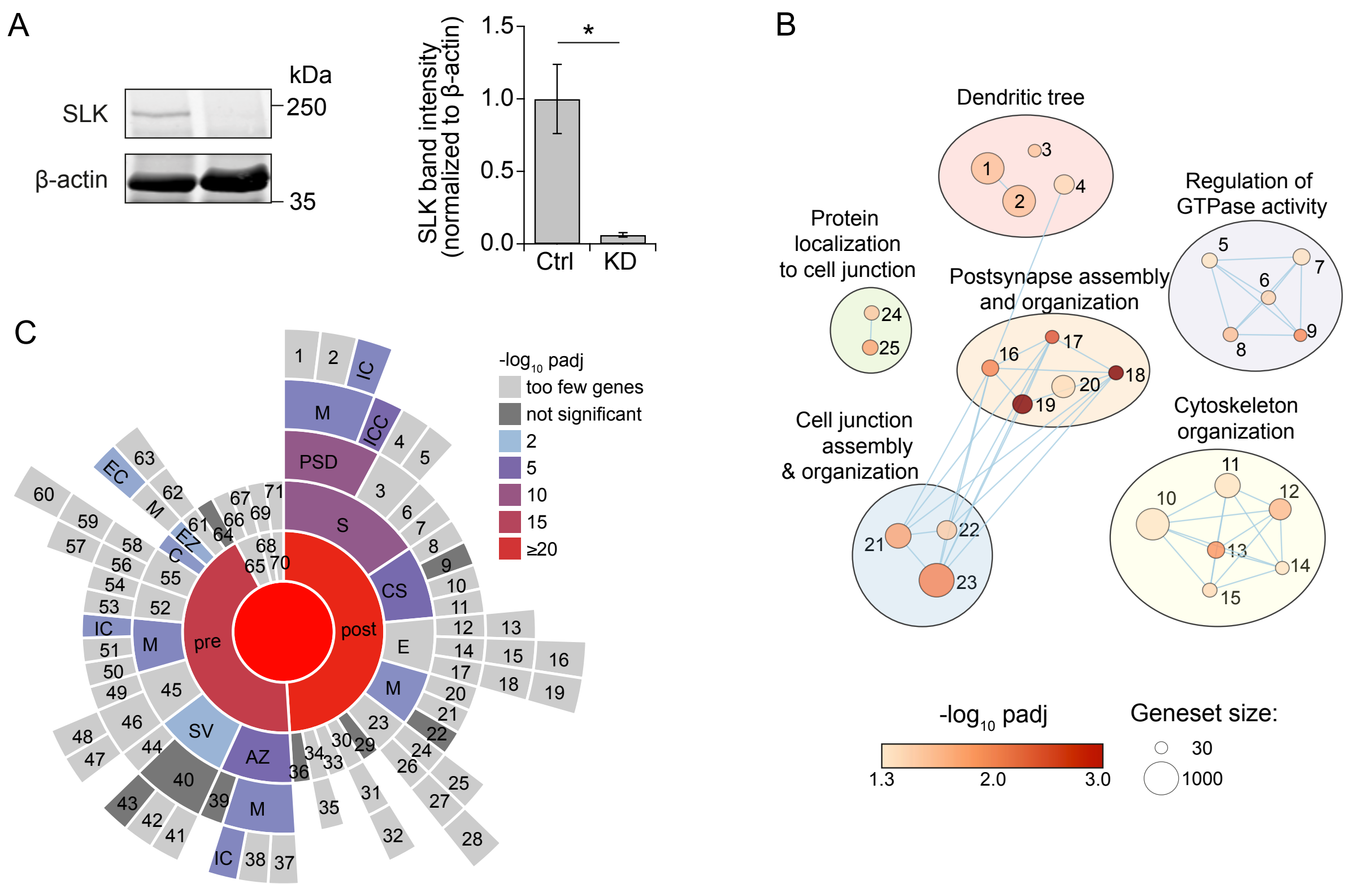

Fig. S1 Identification of the phosphorylation landscape after SLK KD. **A** Knockdown efficiency of the lentiviral shSLK constructs in cultured cortical neurons was verified by immunoblotting against SLK and β-actin. The knockdown reduced SLK levels on average by 93%. n = 5 independent experiments. Unpaired t-test with Welch's correction. **B** Annotated diagram from Fig. 2C.

Code	Ontology term name
	- Dendritic tree
1	○ Dendrite (1)
2	○ Dendritic tree (2)
3	○ Positive regulation of cilium assembly (3)
4	○ Dendrite development (4)
	- Regulation of GTPase activity
5	○ Regulation of GTPase activity (5)
6	○ Positive regulation of GTPase activity (6)
7	○ Regulation of hydrolase activity (7)
8	○ Positive regulation of hydrolase activity (8)
9	○ Activation of GTPase activity (9)
	- Cytoskeleton organization
10	○ Supramolecular fiber organization (10)
11	○ Regulation of cytoskeleton organization (11)
12	○ Regulation of supramolecular fiber organization (12)
13	○ Regulation of protein polymerization (13)
14	○ Regulation of microtubule polymerization (14)
15	○ Microtubule polymerization (15)
	- Postsynapse assembly and organization
16	○ Regulation of postsynapse organization (16)
17	○ Regulation of postsynapse assembly (17)
18	○ Postsynapse assembly (18)
19	○ Postsynapse organization (19)
20	○ Postsynaptic density (20)

- Cell junction assembly and organization
    - 21 Cell junction assembly (21)
    - 22 Regulation of cell junction assembly (22)
    - 23 Cell junction organization (23)
- Protein localization to cell junction
    - 24 Protein localization to synapse (24)
    - 25 Protein localization to cell junction (25)

C Annotated SynGo plot from Fig. 2D.

Code — Ontology term name

- **Post** — **Postsynapse**
    - **S** — **Postsynaptic specialization**
        - **PSD** — **Postsynaptic density**
            - **M** — **Postsynaptic density membrane**
                - 1 — Anchored component of postsynaptic density membrane
                - 2 — Extrinsic component of postsynaptic density membrane
                - **IC** — **Integral component of postsynaptic density membrane**
            - **ICC** — **Postsynaptic density, intracellular component**
        - 3 — Postsynaptic specialization membrane
            - 4 — Extrinsic component of postsynaptic specialization membrane
            - 5 — Integral component of postsynaptic specialization membrane
        - 6 — Postsynaptic specialization of symmetric synapse
        - 7 — Postsynaptic specialization, intracellular component
    - **CS** — **Postsynaptic cytoskeleton**
        - 8 — Postsynaptic actin cytoskeleton
        - 9 — Postsynaptic intermediate filament cytoskeleton
        - 10 — Postsynaptic spectrin-associated cytoskeleton
        - 11 — Postsynaptic septin cytoskeleton
    - **E** — **Postsynaptic endosome**
        - 12 — Postsynaptic endosome membrane
            - 13 — Extrinsic component of postsynaptic endosome membrane
        - 14 — Postsynaptic recycling endosome
            - 15 — Postsynaptic recycling endosome membrane
                - 16 — Anchored component of postsynaptic recycling endosome membrane
        - 17 — Postsynaptic early endosome
            - 18 — Postsynaptic early endosome membrane
                - 19 — Extrinsic component of postsynaptic early endosome
    - **M** — **Postsynaptic membrane**
        - 20 — Extrinsic component of postsynaptic membrane
        - 21 — Anchored component of postsynaptic membrane
        - 22 — Integral component of postsynaptic membrane
    - 23 — Postsynaptic ER
        - 24 — Postsynaptic ER membrane
            - 25 — Integral component of postsynaptic ER membrane
        - 26 — Postsynaptic SER
            - 27 — Postsynaptic SER membrane
                - 28 — Integral component of postsynaptic SER membrane
    - 29 — Postsynaptic cytosol
    - 30 — Spine apparatus
        - 31 — Spine apparatus membrane
            - 32 — Integral component of spine apparatus membrane
    - 33 — Postsynaptic Golgi apparatus
    - 34 — Postsynaptic endocytic zone
        - 35 — Postsynaptic endocytic zone membrane
    - 36 — Postsynaptic ribosome

- **pre** **Presynapse**
  - **AZ** **Presynaptic active zone**
    - **M** **Presynaptic active zone membrane**
      - 37 Extrinsic component of presynaptic active zone membrane
      - 38 Anchored component of presynaptic active zone membrane
      - **IC** **Integral component of presynaptic active zone membrane**
    - 39 Presynaptic active zone cytoplasmic component
  - **SV** **Synaptic vesicle**
    - 40 Synaptic vesicle membrane
      - 41 Integral component of synaptic vesicle membrane
      - 42 Extrinsic component of synaptic vesicle membrane
      - 43 Anchored component of synaptic vesicle membrane
    - 44 Synaptic vesicle lumen
  - 45 Neuronal dense core vesicle
    - 46 Neuronal dense core vesicle membrane
      - 47 Extrinsic component of neuronal dense core vesicle membrane
      - 48 Integral component of neuronal dense core vesicle membrane
    - 49 Neuronal dense core vesicle lumen
  - **M** **Presynaptic membrane**
    - 50 Extrinsic component of presynaptic membrane
    - 51 Anchored component of presynaptic membrane
    - IC Integral component of presynaptic membrane
  - 52 Presynaptic cytoskeleton
    - 53 Presynaptic actin cytoskeleton
    - 54 Presynaptic intermediate filament cytoskeleton
  - 55 Presynaptic ER
    - 56 Presynaptic ER membrane
      - 57 Integral component of presynaptic ER membrane
    - 58 Presynaptic SER (
      - 59 Presynaptic SER membrane
        - 60 Integral component of presynaptic SER membrane
  - **C** **Presynaptic cytosol**
  - **EZ** **Presynaptic endocytic zone**
    - **M** **Presynaptic endocytic zone membrane**
      - **EC** **Extrinsic component of presynaptic endocytic zone membrane**
  - 61 Presynaptic endosome
    - 62 Presynaptic endosome membrane
      - 63 Integral component of presynaptic endosome membrane
  - 64 Presynaptic ribosome
- 65 Synaptic membrane
  - 66 Extrinsic component of synaptic membrane
  - 67 Integral component of synaptic membrane
- 68 Synaptic cleft
  - 69 Extracellular matrix of synaptic cleft
- 70 Perisynaptic space
  - 71 Perisynaptic extracellular matrix

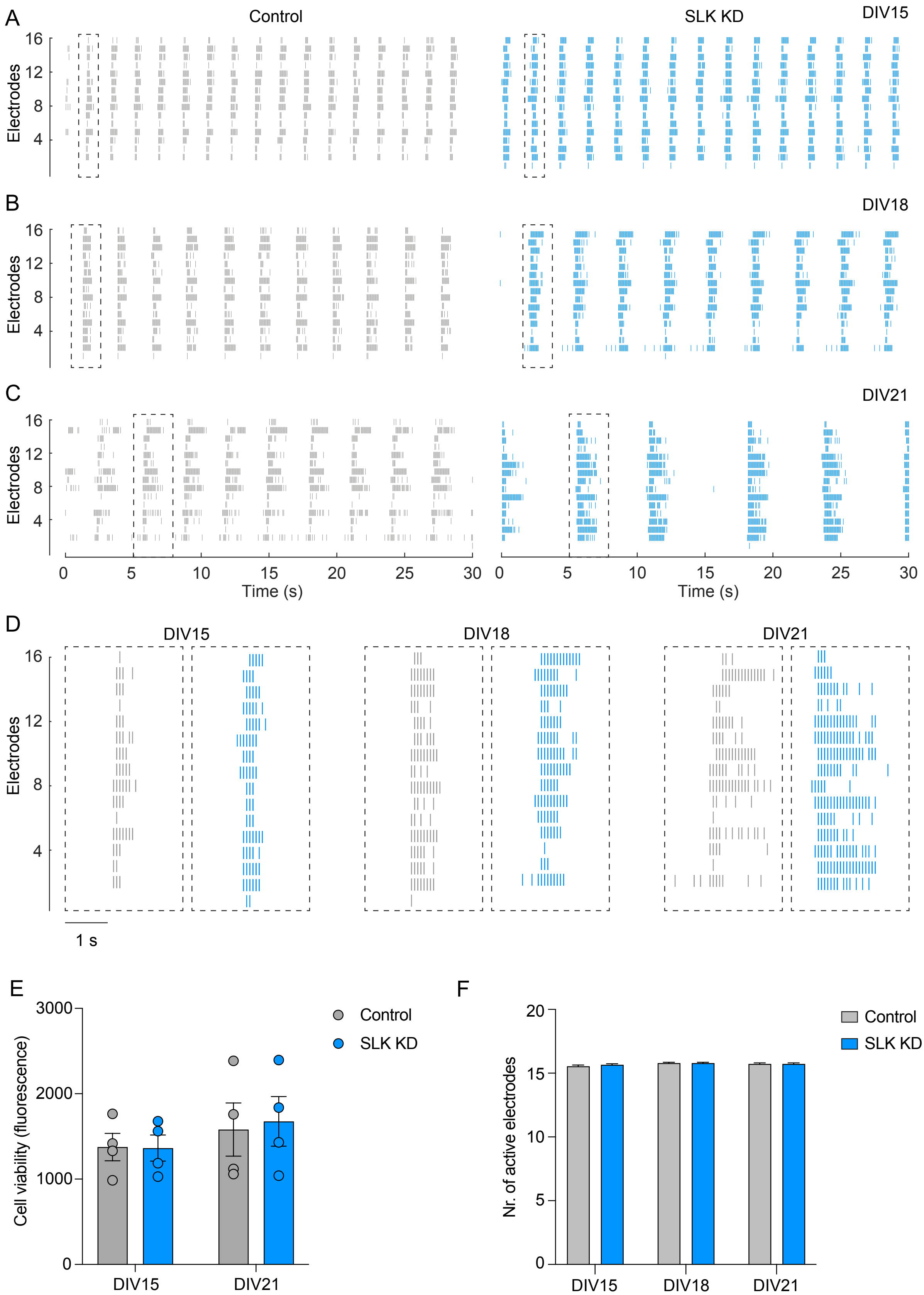
A
Control
SLK KD
DIV15
Electrodes
16
12
8
4
B
DIV18
C
DIV21
0
5
10
15
20
25
30
Time (s)
D
DIV15
DIV18
DIV21
1 s
E
Cell viability (fluorescence)
3000
2000
1000
0
Control
SLK KD
DIV15
DIV21
F
Nr. of active electrodes
20
15
10
5
0
Control
SLK KD
DIV15
DIV18
DIV21

Fig. S2 Alteration of electrophysiological parameters in multi-electrode array recordings after SLK KD in vitro. **A-C** Representative raster spike plots for control and SLK KD neurons on DIV15 (A), 18 (B), and 21 (C). **D** Enlarged views of selected regions from A-C. **E** Neuronal cell viability assessed by the alamarBlue assay was unchanged after SLK KD. n = 4 independent experiments (two-way ANOVA with Holm-Šídák's multiple comparisons test). **F** The number of active electrodes (a proxy for viable, firing neurons) did not differ between control and SLK KD cultures in the MEA experiments, pointing to no differences in seeding density. N = 45-46 wells from 4 independent replicates (two-way ANOVA with Holm-Šídák's multiple comparisons test).

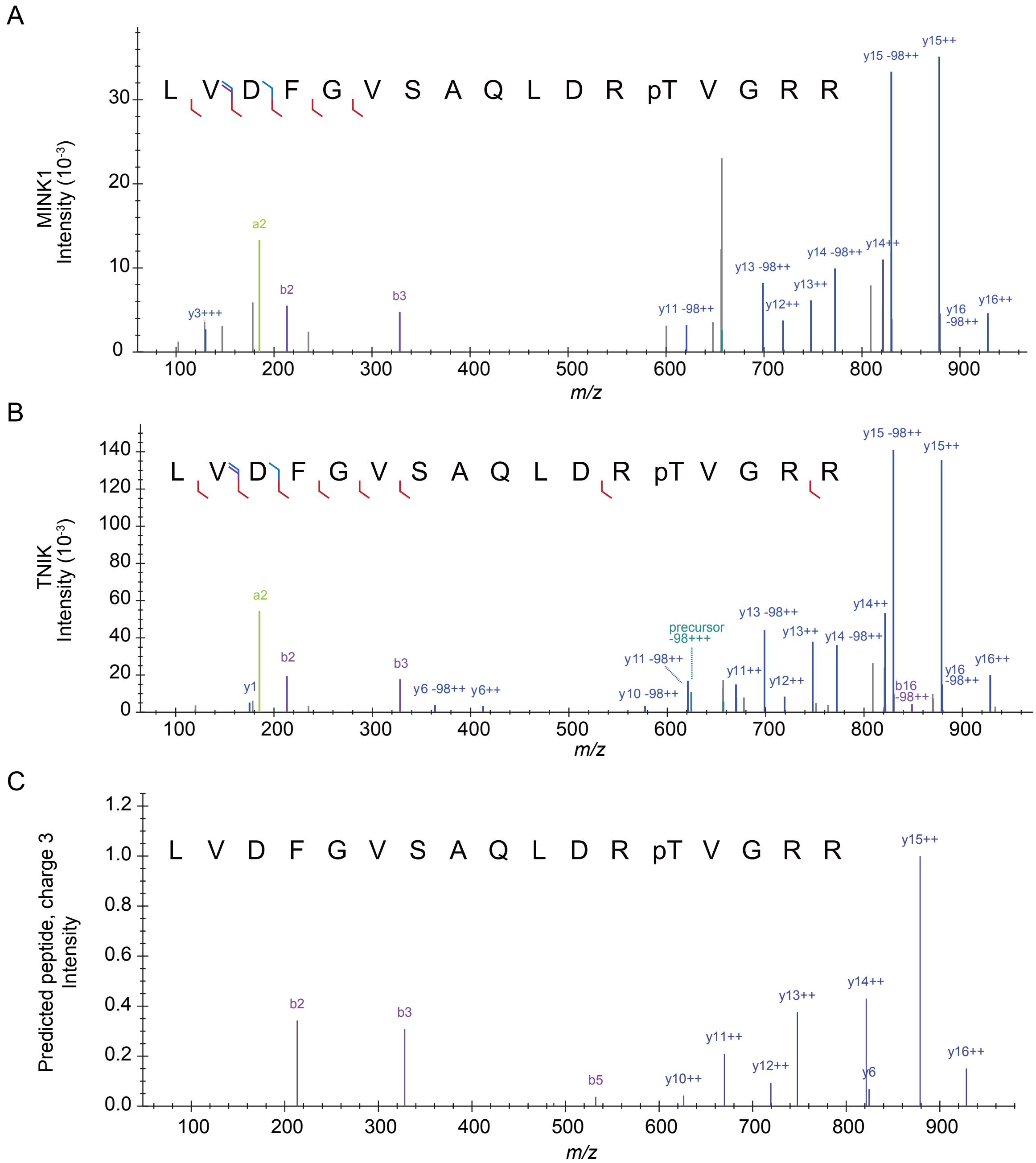

Fig. S3 Mass spectra for *in vitro* kinase reactions **A** Representative mass spectra for the phosphorylated peptide for MINK1. **B** Representative mass spectra for the phosphorylated peptide for TNIK. **C** Predicted mass spectra for the phosphorylated peptide from MINK1 and TNIK.

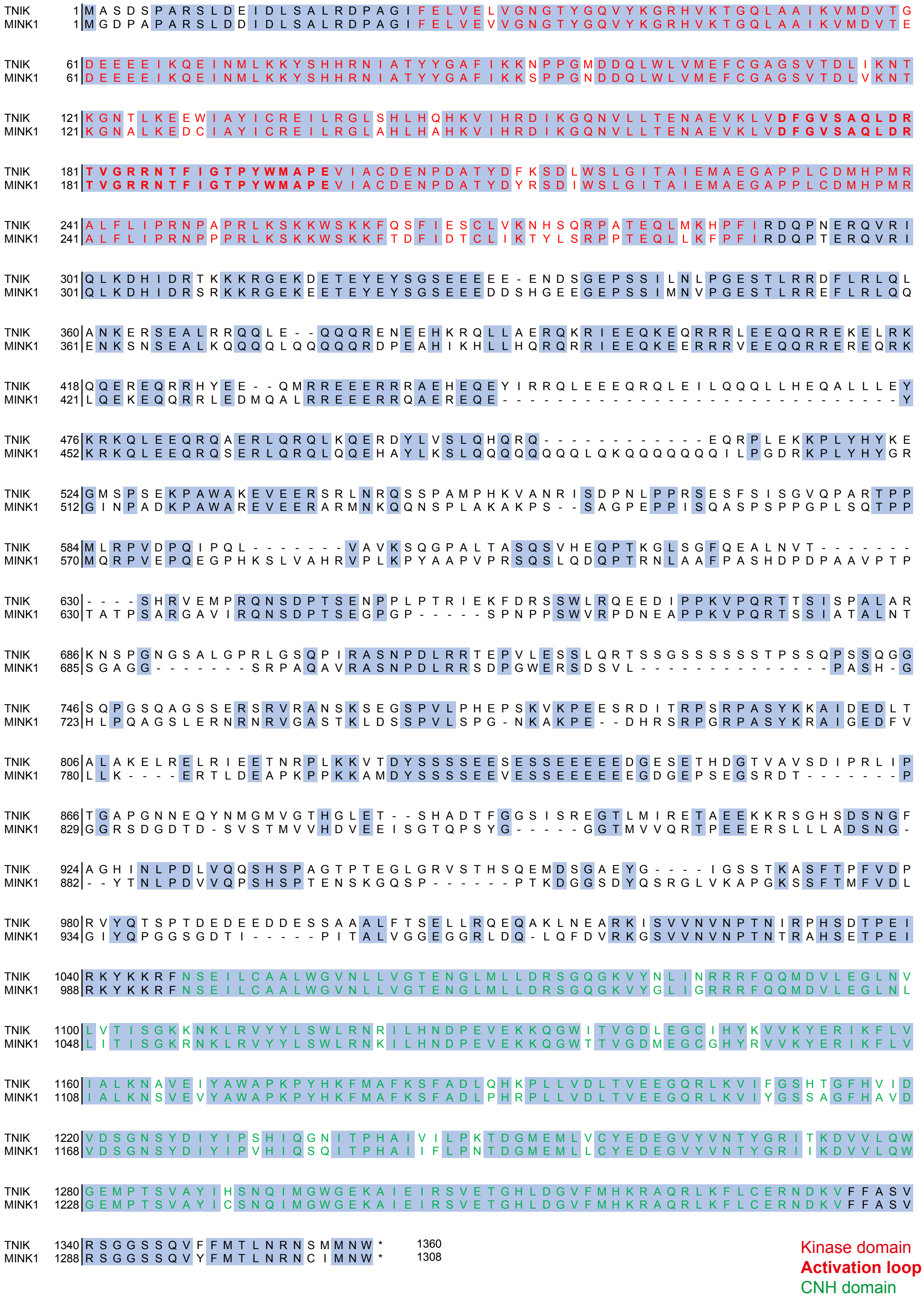

Fig. S4 Comparison of amino acid sequences of MINK1 and TNIK. Mouse MINK1 and TNIK were aligned, and similar structures were highlighted.

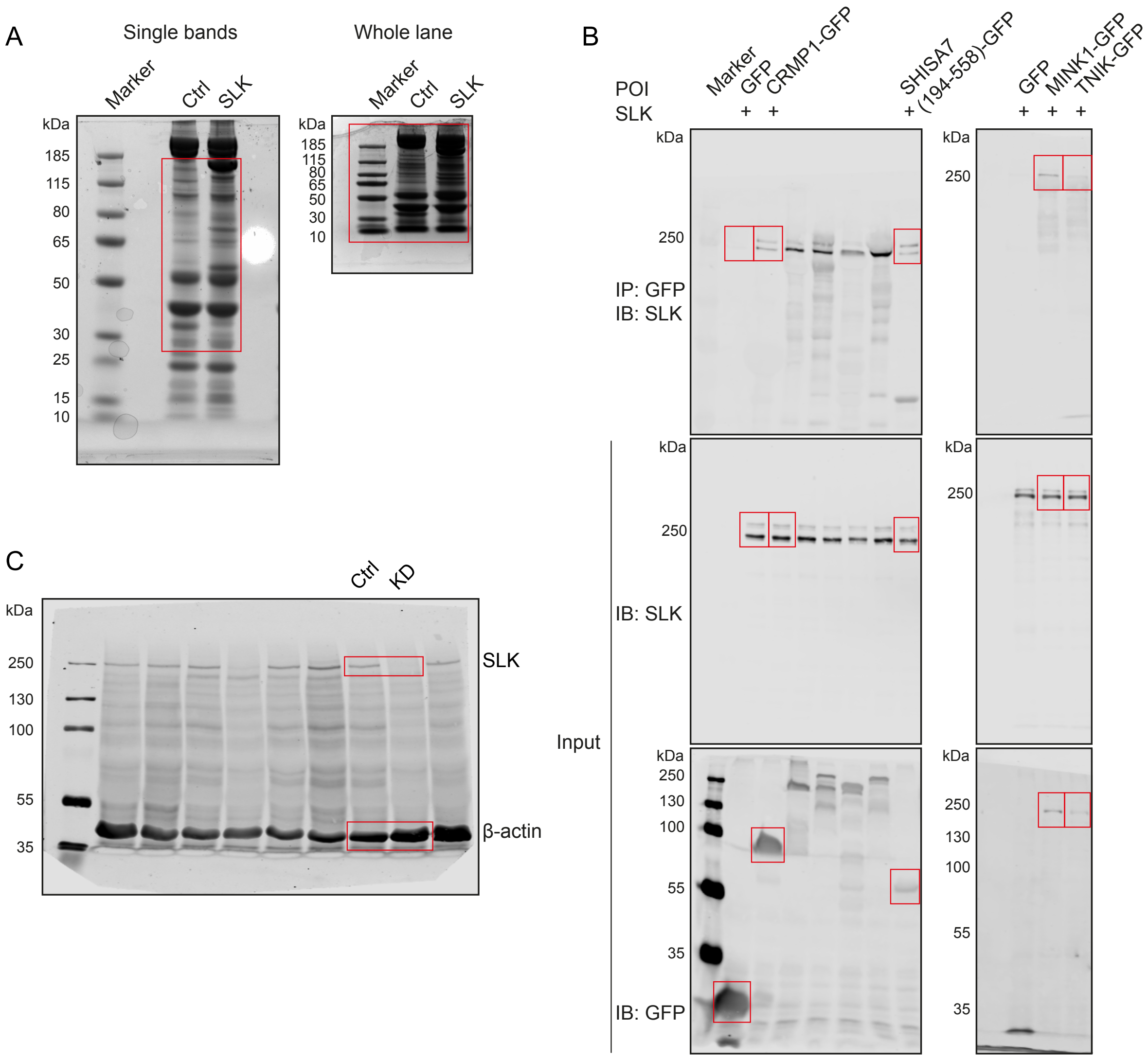

Fig. S5 Uncropped gel images and blots corresponding to Fig. 1B (**A**), Fig. 3D (**B**), and Fig. S1A (**C**). The areas highlighted in red are shown in the corresponding figures.

Table S1 Phosphorylation sites of known SLK substrates (the phosphorylated amino acid is marked in red).

Sequence	Phosphosite	Reference
KAPTARKTTTRRPKP	DCTN1 T145	Zhapparova et al [1]
APTARKTTTRRPKPT	DCTN1 T146	Zhapparova et al [1]
PTARKTTTRRPKPTR	DCTN1 T147	Zhapparova et al [1]
QGRDKYKTLRQIRQG	EZR T567	Machicoane et al [2]
LGRDKYKTLRQIRQG	MSN T558	Machicoane et al [2]
PRFSIAPSSLDPSNR	PLK1 S330	Johnson et al [3]
RFSIAPSSLDPSNRK	PLK1 S331	Johnson et al [3]
APSSLDPSNRKPLTV	PLK1 S335	Johnson et al [3]
YDGERKKTLCGTPNY	PLK1 T210	Johnson et al [3]
ETSCIKETYLRIKKN	PLK1 T259	Johnson et al [3]
IPARLPITCLTIPPR	PLK1 T317	Johnson et al [3]
RLPITCLTIPPRFSI	PLK1 T320	Johnson et al [3]
PSNRKPLTVLNKGLE	PLK1 T341	Johnson et al [3]
SSPQRVTSTQQQTRI	PXN S250	Quizi et al [4]
AGRDKYKTLRQIRQG	RDX T564	Machicoane et al [2]
ARRGKKKSGCLILXX	RHOA S188	Guillluy et al [5]
VSAKNTRTIQRRDSF	SLK T183	Pike et al [6]
RTIQRRDSFIGTPYW	SLK T189	Pike et al [6]

References

1. Zhapparova ON, Fokin AI, Vorobyeva NE, Bryantseva SA, Nadezhdina ES. Ste20-like protein kinase SLK (LOSK) regulates microtubule organization by targeting dynactin to the centrosome. Mol Biol Cell. 2013;24:3205–3214.
2. Machicoane M, de Frutos CA, Fink J, Rocancourt M, Lombardi Y, Gare S, Piel M, Echard A. SLK-dependent activation of ERMs controls LGN-NuMA localization and spindle orientation. J Cell Biol. 2014;205:791–799.
3. Johnson TM, Antrobus R, Johnson LN. Plk1 activation by Ste20-like kinase (Slk) phosphorylation and polo-box phosphopeptide binding assayed with the substrate translationally controlled tumor protein (TCTP). Biochemistry. 2008;47:3688–3696.
4. Quizi JL, Baron K, Al-Zahrani KN, O'Reilly P, Sriram RK, Conway J, Laurin AA, Sabourin LA. SLK-mediated phosphorylation of paxillin is required for focal adhesion turnover and cell migration. Oncogene. 2013;32:4656–4663.
5. Guilluy C, Rolli-Derkinderen M, Loufrani L, Bourgá A, Henrion D, Sabourin L, Loirand G, Pacaud P. Ste20-related kinase SLK phosphorylates Ser188 of RhoA to induce vasodilation in response to angiotensin II Type 2 receptor activation. Circ Res. 2008;102:1265–1274.
6. Pike ACW, Rellos P, Niesen FH, Turnbull A, Oliver AW, Parker SA, Turk BE, Pearl LH, Knapp S. Activation segment dimerization: a mechanism for kinase autophosphorylation of non-consensus sites. EMBO J. 2008;27:704–714.
